# Analysis of sandwich graphene origami composite plate sandwiched by piezoelectric/piezomagnetic layers: A higher-order electro-magneto-elastic analysis

**DOI:** 10.1016/j.heliyon.2024.e29436

**Published:** 2024-04-12

**Authors:** Thaier J. Ntayeesh, Mohammad Arefi

**Affiliations:** a Faculty of Mechanical Engineering, College of Engineering, University of Baghdad, Baghdad, 10071, Iraq; b Faculty of Mechanical Engineering, Department of Solid Mechanics, University of Kashan, Kashan, 87317-51167, Iran

**Keywords:** Piezoelectric/piezomagnetic layers, Initial electromagnetic loads, Sandwich graphene origami composite plate, Thickness stretched plate, Micromechanical model, Volume fraction, Folding degree, Temperature

## Abstract

This work applies a higher order thickness-stretched model for the electro-elastic analysis of the composite graphene origami reinforced square plate sandwiched by the piezoelectric/piezomagnetic layers subjected to the thermal, electric, magnetic and mechanical loads. The plate is manufactured of a copper matrix reinforced with graphene origami where the effective material properties are calculated based on the micromechanical models as a function of volume fraction and folding degree of graphene origami, material properties of matrix, reinforcement, and local temperature. The governing equations are derived using the virtual work principle in terms of the bending, shear and stretching functions, in-plane displacements, electric, and magnetic potentials. The numerical results including various displacement components, maximum electric, and magnetic potentials are presented with changes of volume fraction, folding degree of reinforcement, electrical, magnetic, and thermal loading. A verification investigation is presented for approve of the methodology, and the solution procedure. The main novelty of this work is simultaneous effect of the thickness stretching and the multi-field loading on the electromagnetic bending results of the sandwich plate. Another novelty of this work is usage of graphene origami nano-reinforcement as a controllable material in a sandwich structure subjected to multi-field loadings. The results show an increase in bending, shear, and stretching deflections with an increase in electromagnetic loads, and folding degree as well as a decrease in volume fraction of reinforcement.

## 1 Introduction

The piezoelectric/piezomagnetic materials are extensively used in the electro-magneto-mechanical system in various scales for performing a definite work or sensing and measuring the deformation or stress. These materials may be used in various systems as sensor and actuator [[1], [2], [3]]. Combination of these materials in various situations with different material composition presents the novel materials and structures [[4], [5], [6], [7]]. This paper is organized to suggest a new magneto-electro-mechanical sandwich composite plate composed of the graphene origami reinforced plate sandwiched by piezoelectric/piezomagnetic layers in the electro-magneto-thermo-mechanical environment. The Introduction section and literature review is presented with studying the more related papers on the nanomaterials and nanostructures, piezoelectric materials and structures, and higher-order shear deformable models.

To analyze a new smart system using a higher order deformable model, Zhang et al. [8] developed the three lamination patterns and viscoelasticity for the finite element analysis of smart piezoelectric shells and plates based on the zig zag and Mindlin theories. A 7th degree of freedom model was used for finite element formulation of the hybrid problem. The numerical results have been compared with the finite element results at first stage and finally with analytical approach. In another work, a sandwich hybrid model was extended for investigating effect of initial electric potentials on the nonlinear responses of the piezoelectric structures. Azrar et al. [9] developed a modal analysis for the sandwich piezoelectric beam based on linear and nonlinear analyses subjected to applied voltage. Using some assumptions and simplifications, the various diagrams such as load-/amplitude,/frequency and amplitude-nonlinear frequency were depicted with changes of applied voltage. Production of novel three-dimensional nanostructures and nanosheets using some chemical process and its application in various situations was studied by various researchers. For example, Tang et al. [10] summarized advantages of 3D structures in nanoscales. Recently, material scientists developed the bilayer structures in origami configuration with higher responsive properties. Furthermore, it was mentioned preserving the deformability without supplying the additional energy using the origami architectural structures applicable in the aerospace vehicles, bio-medical aims, and devices in micro-fluid systems. The folding process is used as the result of hydrogenation process for manufacturing three-dimensional graphene sheets with flexible material properties. For example, Joung et al. [11] developed a folding process for production of graphene oxide using polyhedral in order to control geometric characteristics of graphene origami as a folded structure. They demonstrated some novel properties of invented three dimensional folded structures such as optical switching and water permeable. In another work, a solar-based generator using steam and three dimensional graphene oxide was suggested by Wang et al. [12]. Application of the proposed three-dimensional structure was illustrated in vapor evaporation, water transportation, and sun-light absorption. Zhang et al. [13] characterized application of graphite materials for manufacturing the dual ion batteries in a metal-based constituent. To exhibit advantages of the graphene origami in various situations because of novel electro-optic/magneto-mechanical properties, Chen et al. [14] provided the experimental work on the origami/kirigami. Review on the recent work in the origami structures indicates that these materials can be used in biological and biomedical applications. Enabling the biological and optical properties of micro and nano scale kirigami and origami using three dimensional folding process was illustrated in this paper. Furthermore, the new devices made of three-dimensional nanostructures were suggested by some researchers in the recent works. Lam et al. [15] described characteristics of graphene origami applicable as a rotary nanomotor composed of double walled carbon nanotubes. The nanomotor was fabricated from attaching the graphene origami into a long tube as a rotor. The results of the experimental work were justified through comparison with molecular dynamic results. Drug delivery application of the graphene origami in the small scales was developed by Luo et al. [16] for application in the bio-medical devices using the molecular dynamic based analysis. The complex pattern of the graphene origami was explained using the hydrogenation and arranging the hydrogen atoms inside the folded graphene. Due to manufacturing of these materials in a bio-based polymer, one can suggest these composed structure for application in the bio-sensors and micro-fluidic instruments. Details of three operations named as pasting, folding and bending in order to functionalize folded structures have been illustrated by Lu et al. [17] using the clay sheets or carbon nanofillers.

The sandwich and multi-layered structures can be used in various situations such as electromechanical and electromagnetomechanical systems. The integrated piezoelectric layers can be used for sensing the deformations or performing a definite work. Shuyu [18] analyzed various characteristics of an ultrasonic sandwich transducer using equivalent circuit method subjected to liquid and solid loads as application in the drilling and machining. Resonance frequency analysis was investigated with changes of structure as well as load. Wang et al. [19] presented the dynamic analysis on the wave propagation in a sandwich transducer made of the piezoelectric materials and an optimization study in order to improve output performance. The bending and free vibration characteristics of sandwich transducer were presented for PZT element. The optimization of shapes and sizes was presented as main conclusion of this work. Novel conductivity properties using the laser-based printing technology was explained by Zang et al. [20] to cover an acceptable electromagnetic interference in the foldable structures. The capability of this proposed structure was illustrated in the rough environments. Manufacturing the folded structure in a polystyrene matrix was proposed by Basu et al. [21]. A new concept regarding physical neutral surface was employed by researcher for analysis of a sandwich piezoelectric plate [22]. Li et al. [23] summarized heat dissipation capability of the carbon films in various shapes in a polyimide matrix using high temperature annealing process. From the materials science and various chemical processes, one can study effect of various shapes of graphene and nanostructures on the structural behaviors of nanocomposite structures. Tomioka et al. [24] studied effect of various shapes of graphene sheets in wavy format on the impact results of carbon nanotubes using molecular dynamic scheme. Fan et al. [25] investigated the effect of required parameters for control of hydrogenation process in preparation of kirigami/origami in order to optimum operation and reflection of better mechanical, optical and electronic properties. White et al. [26] demonstrated application of the nanosize graphene structures in DNA. Impact of the electric potential was studied on the dynamical behavior of lower-order beam was studied by Wang et al. [27]. It was concluded that the piezoelectric layer's position has a significant effect on the vibration frequency and mode shape. Lin et al. [28] suggested an ultrasonic transducer made of smart materials. The structure was fabricated from thin hollow disks. After propose of two sandwich transducers, the results of these experimental work was confirmed through comparison with theoretical results in literature. Konka et al. [29] developed an experimental study to present various analysis such strength, bending and vibration of the piezoelectric composite structures. The effect of various material characteristics of piezoelectric composite structure was studies on the responses. An atomic force microscope was employed by Ebbesen and Hiura [30] in a graphite structure. Graphene origami materials reflects an extended range for material properties in the thermal environment. As instance, an improved and extended range of negative to positive thermal expansion coefficient was used by Ho et al. [31] using the graphene origami. Because of higher flexibility of the graphene origami materials, there are some applications of these materials in the different structures with energy saving and absorption purposes. A compressive modeling was used for investigating energy absorption of Miura-ori reinforced polymer based matrix [32]. A detailed study on the folding manufacturing process was explained with focus on the three applicable methods [33]. Lu et al. [34] explained usage of the PCM nanowire embedded into a nano-photonic circuit to study switching dynamics in mixed-mode operation.

Some works on the emerging properties of folding process and graphene origami as well detailed material properties were reported in the detail [[35], [36], [37]]. The important equations for constitutive relations and verification study may be observed in References [[38], [39], [40], [41], [42], [43], [44], [45]]. Yang et al. [46] introduced a new material with flexible properties reflecting electro-magnetic properties in thermal environment. Yue et al. [47] reported a review paper on the application of sandwich structures, and materials, smart, and piezoelectric structures and other novel structures. The advanced methods and models for analysis of aforementioned structures were developed in this review paper. Porosity property may be used in engineering structures because of high strength and low density as reported in the recent works [[48], [49], [50], [51], [52]]. Cai et al. [53] extended a new work on the tunability of the graphene origami. They have shown details of tunability such as negative value of thermal expansion coefficient and Poisson's ratio. The mentioned tunability was obtained with adjusting the geometric parameters of the Miura-ori graphene origami. The improvement of the structural behavior such as strength, density, Poisson's ratio and thermal expansion coefficient was justified through comparison between the molecular dynamic simulation results and the continuum mechanics analysis as a function of small scale. Chen et al. [54] summarized excellent behavior of the graphene nano structures fabricated from rolled or folded shapes of graphene in mono or bi-layers conditions which reflects various interesting properties in thermal, mechanical and electrical scopes. They have shown that geometric parameters of the folded graphene such as folding angle leads to high tunability of structure. Finally, it was concluded that graphene origami auxetic metamaterials are an excellent scheme to arrive an atomically tunable and precise nano structure.

Araki and Zhang [55] summarized importance of the graphene origami because of its negative thermal expansion in correction of the optical properties of the structures made of it. Furthermore, some applications of this material in plasmon polaritons were developed. There are some applications of the origami graphene in emission as well as optical absorption in surface with photonic properties [56]. The new higher-order shear deformation theories have been proposed by various researchers in order to arrive at more accurate formulation of the structures. Bennoun et al. [57] studied free vibration responses of a functionally graded sandwich square plate based on five-variable plate theory. Accuracy of this theory was justified through comparison of the present results with literature. In order to account stretching along the thickness direction and obtain more general formulation, the higher-order thickness-stretchable kinematic model was used in the recent papers. The effect of different and combined boundary conditions was studied on the bending responses using an analytical method [58]. Deng et al. [59] developed application of the piezoelectric materials in sandwich configuration for small scale resolution and higher scan rate. The main importance and difference of this work was high resonance frequency of the proposed structure than previous actuators. Huang et al. [60] studied vibration control of smart sandwich plate made of the piezoelectric materials as face sheets. The motion's equations were derived using the Hamilton's principle and the solution was proposed using Rayleigh-Ritz approach. It was arrived best effect of active control using the larger coefficient of speed feedback. Quan et al. [61] used an auxetic material for nonlinear dynamic and vibration responses of the piezoelectric sandwich plate subjected to uniform pressure based on the Galerkin method and 4th order Runge–Kutta. The effect of imperfection was studied on the nonlinear dynamic responses of sandwich piezoelectric auxetic plate. Compressive loading analysis and energy absorption capacity of a metamaterial structure in the Miura-ori configuration was studied experimentally and numerically [62,63]. Agricultural application of the graphene origami was explained in the literature [64,65]. The effect of out of plane shear and normal loading was studied on the nonlinear responses of composite plates using the higher-order shear deformation theory [66]. Concurrent effect of piezoelasticity effect and flexibility was studied on the active vibration control of cantilever beam using the Hamilton's principle [67,68]. The effect of the carbon nanofillers in a polymer matrix was studied on the elastic static bending responses of the cylindrical panels with variable thickness [69]. Thermal properties of composite materials made of nanomaterial reinforcement was explained in the works [70,71]. The micro structure dependent properties of novel composite materials and structures was explained in the recent works [[72], [73], [74], [75], [76]].

A review on the recent works on the graphene origami, piezoelectric/piezomagnetic materials, nanofillers, size dependent analysis, higher-order shear and normal deformation theory and thickness stretching concept was completed above. One can see that there is no available published work on the multi-field bending responses of sandwich graphene origami reinforced sandwich plate integrated with the piezoelectric/piezomagnetic layers based on a thickness stretched model. In this paper, a higher-order thickness-stretched model based on higher-order shear and normal deformation theory is developed for electro-magneto-thermal analysis of the sandwich plate, where the effective material properties of the core are estimated using Halpin-Tsai micromechanical model. The results will be presented with changes of material characteristics of graphene origami and multi filed loading characteristics. The proposed structure in this paper may be used as an intelligent system in defence devices and equipment because of tunable material properties and responses.

## 2 Higher-order analysis

The governing equations and the constitutive relations are developed in this section based on the thickness stretched model and the magneto-electro-elastic relations. The constitutive relations based on the magneto-electro-elastic relations in three dimensional coordinate system excited by a three dimensional magnetic and electric potentials are defined as follows:

(1) $$\left\{\begin{array}{c} \sigma_{x}\\ \sigma_{y}\\ \sigma_{z}\\ \sigma_{yz}\\ \sigma_{xz}\\ \sigma_{xy} \end{array}\right\}=\left[\begin{array}{ccc} \begin{array}{c} \begin{array}{cc} C_{11}^{P} & C_{12}^{P} \end{array}\\ \begin{array}{c} \begin{array}{cc} C_{12}^{P} & C_{22}^{P} \end{array}\\ \begin{array}{cc} C_{13}^{P} & C_{23}^{P} \end{array} \end{array} \end{array} & \begin{array}{c} C_{13}^{P}\\ \begin{array}{c} C_{23}^{P}\\ C_{33}^{P} \end{array} \end{array} & \begin{array}{ccc} 0 & 0 & 0\\ 0 & 0 & 0\\ 0 & 0 & 0 \end{array}\\ \begin{array}{c} \begin{array}{cc} 0 & 0 \end{array}\\ \begin{array}{c} \begin{array}{cc} 0 & 0 \end{array}\\ \begin{array}{cc} 0 & 0 \end{array} \end{array} \end{array} & \begin{array}{c} 0\\ \begin{array}{c} 0\\ 0 \end{array} \end{array} & \begin{array}{ccc} C_{44}^{P} & 0 & 0\\ 0 & C_{55}^{P} & 0\\ 0 & 0 & C_{66}^{P} \end{array} \end{array}\right]\left\{\begin{array}{c} \varepsilon_{x}-\alpha T\\ \varepsilon_{y}-\alpha T\\ \varepsilon_{z}-\alpha T\\ \gamma_{yz}\\ \gamma_{xz}\\ \gamma_{xy} \end{array}\right\}-\left[\begin{array}{ccc} 0 & 0 & e_{13}\\ 0 & 0 & e_{23}\\ \begin{array}{c} 0\\ 0\\ \begin{array}{c} e_{51}\\ 0 \end{array} \end{array} & \begin{array}{c} 0\\ e_{42}\\ \begin{array}{c} 0\\ 0 \end{array} \end{array} & \begin{array}{c} e_{33}\\ 0\\ \begin{array}{c} 0\\ 0 \end{array} \end{array} \end{array}\right]\left\{\begin{array}{c} E_{x}\\ E_{y}\\ E_{z} \end{array}\right\}-\left[\begin{array}{ccc} 0 & 0 & q_{31}\\ 0 & 0 & q_{32}\\ \begin{array}{c} 0\\ 0\\ \begin{array}{c} q_{15}\\ 0 \end{array} \end{array} & \begin{array}{c} 0\\ q_{24}\\ \begin{array}{c} 0\\ 0 \end{array} \end{array} & \begin{array}{c} q_{33}\\ 0\\ \begin{array}{c} 0\\ 0 \end{array} \end{array} \end{array}\right]\left\{\begin{array}{c} H_{x}\\ H_{y}\\ H_{z} \end{array}\right\}$$

where in Eq. (1) the electric $E_{i}$ and magnetic $H_{i}$ fields are defined using electric $\Psi =\frac{2z}{h}\Psi_{0}-\Psi \left(x,y\right)\cos\frac{\pi z}{h}$ and magnetic $\Phi =\frac{2z}{h}\Phi_{0}-\Phi \left(x,y\right)\cos\frac{\pi Z}{h}$ potentials as in Eq. (2):

$$E_{x}=\frac{\partial \Psi }{\partial x}\cos\frac{\pi z}{h},E_{y}=\frac{\partial \Psi }{\partial y}\cos\frac{\pi z}{h},E_{z}=-\frac{2}{h}\Psi_{0}-\frac{\pi }{h}\Psi \;\sin\frac{\pi z}{h}$$

(2) $$H_{x}=\frac{\partial \Phi }{\partial x}\cos\frac{\pi z}{h},H_{y}=\frac{\partial \Phi }{\partial y}\cos\frac{\pi z}{h},H_{z}=-\frac{2}{h}\Phi_{0}-\frac{\pi }{h}\Phi \;\sin\frac{\pi z}{h}$$

Eqs. (3), (4) presents the electric and magnetic-displacement relations as [[72], [73], [74], [75], [76]]:

(3) $$\left\{\begin{array}{c} D_{x}\\ D_{y}\\ D_{z} \end{array}\right\}=\left[\begin{array}{ccc} \begin{array}{cc} 0 & 0 \end{array} & 0 & \begin{array}{ccc} 0 & e_{15} & 0 \end{array}\\ \begin{array}{cc} 0 & 0 \end{array} & 0 & \begin{array}{ccc} e_{24} & 0 & 0 \end{array}\\ \begin{array}{cc} e_{31} & e_{32} \end{array} & e_{33} & \begin{array}{ccc} 0 & 0 & 0 \end{array} \end{array}\right]\left\{\begin{array}{c} \varepsilon_{x}-\alpha T\\ \varepsilon_{y}-\alpha T\\ \varepsilon_{z}-\alpha T\\ \gamma_{yz}\\ \gamma_{xz}\\ \gamma_{xy} \end{array}\right\}+\left[\begin{array}{ccc} \eta_{11} & 0 & 0\\ 0 & \eta_{22} & 0\\ 0 & 0 & \eta_{33} \end{array}\right]\left\{\begin{array}{c} E_{x}\\ E_{y}\\ E_{z} \end{array}\right\}+\left[\begin{array}{ccc} d_{11} & 0 & 0\\ 0 & d_{22} & 0\\ 0 & 0 & d_{33} \end{array}\right]\left\{\begin{array}{c} H_{x}\\ H_{y}\\ H_{z} \end{array}\right\}$$

And

(4) $$\left\{\begin{array}{c} B_{x}\\ B_{y}\\ B_{z} \end{array}\right\}=\left[\begin{array}{ccc} 0 & \begin{array}{cc} 0 & 0 \end{array} & \begin{array}{ccc} 0 & q_{15} & 0 \end{array}\\ 0 & \begin{array}{cc} 0 & 0 \end{array} & \begin{array}{ccc} q_{24} & 0 & 0 \end{array}\\ q_{31} & \begin{array}{cc} q_{32} & q_{33} \end{array} & \begin{array}{ccc} 0 & 0 & 0 \end{array} \end{array}\right]\left\{\begin{array}{c} \varepsilon_{x}-\alpha T\\ \varepsilon_{y}-\alpha T\\ \varepsilon_{z}-\alpha T\\ \gamma_{yz}\\ \gamma_{xz}\\ \gamma_{xy} \end{array}\right\}+\left[\begin{array}{ccc} d_{11} & 0 & 0\\ 0 & d_{22} & 0\\ 0 & 0 & d_{33} \end{array}\right]\left\{\begin{array}{c} E_{x}\\ E_{y}\\ E_{z} \end{array}\right\}+\left[\begin{array}{ccc} \mu_{11} & 0 & 0\\ 0 & \mu_{22} & 0\\ 0 & 0 & \mu_{33} \end{array}\right]\left\{\begin{array}{c} H_{x}\\ H_{y}\\ H_{z} \end{array}\right\}$$

**Fig. 1:**
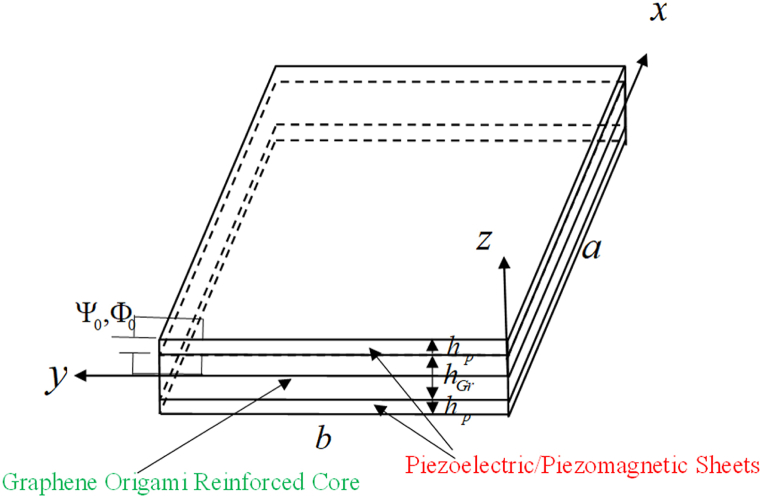
The schematic figure of a sandwich piezoelectric plate made of graphene origami.

The governing relations for the graphene origami reinforced core are presented as follows (Fig. 1):

(5) $$\begin{array}{c} \left\{\begin{array}{c} \sigma_{x}\\ \sigma_{y}\\ \sigma_{z}\\ \sigma_{yz}\\ \sigma_{xz}\\ \sigma_{xy} \end{array}\right\}=\left[\begin{array}{ccc} \begin{array}{c} \begin{array}{cc} C_{11} & C_{12} \end{array}\\ \begin{array}{c} \begin{array}{cc} C_{12} & C_{22} \end{array}\\ \begin{array}{cc} C_{13} & C_{23} \end{array} \end{array} \end{array} & \begin{array}{c} C_{13}\\ \begin{array}{c} C_{23}\\ C_{33} \end{array} \end{array} & \begin{array}{ccc} 0 & 0 & 0\\ 0 & 0 & 0\\ 0 & 0 & 0 \end{array}\\ \begin{array}{c} \begin{array}{cc} 0 & 0 \end{array}\\ \begin{array}{c} \begin{array}{cc} 0 & 0 \end{array}\\ \begin{array}{cc} 0 & 0 \end{array} \end{array} \end{array} & \begin{array}{c} 0\\ \begin{array}{c} 0\\ 0 \end{array} \end{array} & \begin{array}{ccc} C_{44} & 0 & 0\\ 0 & C_{55} & 0\\ 0 & 0 & C_{66} \end{array} \end{array}\right]\left\{\begin{array}{c} \varepsilon_{x}-\alpha T\\ \varepsilon_{y}-\alpha T\\ \varepsilon_{z}-\alpha T\\ \gamma_{yz}\\ \gamma_{xz}\\ \gamma_{xy} \end{array}\right\}\text{,} \end{array}$$

In which in Eq. (5), $C_{ij}$ are stiffness coefficients, $\alpha_{eff}$ is effective thermal expansion, T is thermal loading, $\sigma_{ij}$ are stress components, and $\varepsilon_{i},\gamma_{ij}$ are normal and shear strain components. $C_{ij}$ can be obtained in terms of E and ν for three dimensional analysis. The effective E, ν and α of the shell are assumed using Eq. (6) as follows [38,77]:

$$E=\frac{1+\xi \eta V_{Gr}}{1-\eta V_{Gr}}E_{Cu}f_{E}\left(H_{Gr},V_{Gr},T\right)\text{,}$$

$$\nu =\left(\nu_{Gr}V_{Gr}+\nu_{Cu}V_{Cu}\right)f_{\nu }\left(H_{Gr},V_{Gr},T\right)\text{,}$$

(6) $$\alpha =\left(\alpha_{Gr}V_{Gr}+\alpha_{Cu}V_{Cu}\right)f_{\alpha }\left(H_{Gr},V_{Gr},T\right)\text{,}$$

in which, $\eta =\frac{\frac{E_{Gr}}{E_{Cu}}-1}{\frac{E_{Gr}}{E_{Cu}}+\xi },\xi =2\frac{l_{Gr}}{t_{Gr}}$, and $V_{Cu},V_{Gr}$ are used as content percentage of Cu as matrix and graphene origami as reinforcement. Furthermore, $\left(\nu_{Gr},\nu_{Cu}\right)$ and $\left(\alpha_{Gr},\alpha_{Cu}\right)$ are Poisson's ratio and thermal expansion coefficient of graphene origami and Cu matrix, respectively. The relation between volume fractions of graphene and cooper matrix are developed as $V_{Gr}=\frac{W_{Gr}}{W_{Gr}+\frac{\rho_{Gr}}{\rho_{Cu}}\left(1-W_{Gr}\right)},V_{Cu}=1-V_{Gr}$.

The modification functions $f_{E},f_{\nu },f_{\alpha }$ are for correction and adoption of the effective material properties using Eq. (7) as follows [38]:

$$f_{E}=1.11-1.22V_{Gr}-0.134\left(\frac{T}{T_{0}}\right)+0.559V_{Gr}\left(\frac{T}{T_{0}}\right)-5.5V_{Gr}H_{Gr}+38V_{Gr}^{2}H_{Gr}-20.6V_{Gr}^{2}H_{Gr}^{2}\text{,}$$

$$f_{\nu }=1.01-1.43V_{Gr}+0.165\left(\frac{T}{T_{0}}\right)-1.1V_{Gr}H_{Gr}\left(\frac{T}{T_{0}}\right)-16.8V_{Gr}H_{Gr}+16V_{Gr}^{2}H_{Gr}^{2}$$

(7) $$f_{\alpha }=0.794-16.8V_{Gr}^{2}-0.0279{\left(\frac{T}{T_{0}}\right)}^{2}+0.182\left(1+V_{Gr}\right)\left(\frac{T}{T_{0}}\right)$$

Where, $T,T_{0}$ are local temperature and reference temperature of environment, respectively.

The kinematic relations including three displacement components (Eq. (8)) are developed as follows:

$$u_{x}\left(x,y,z\right)=u\left(x,y\right)-z\frac{\partial w_{b}\left(x,y\right)}{\partial x}-f\left(z\right)\frac{\partial w_{s}\left(x,y\right)}{\partial x}\text{,}$$

$$v_{y}\left(x,y,z\right)=v\left(x,y\right)-z\frac{\partial w_{b}\left(x,y\right)}{\partial y}-f\left(z\right)\frac{\partial w_{s}\left(x,y\right)}{\partial y}$$

(8) $$u_{z}\left(x,y,z\right)=w_{b}\left(x,y\right)+w_{s}\left(x,y\right)+g\left(z\right)\chi \left(x,y\right)\text{,}$$

Where the stretching, shear and bending deflections are denoted by $\chi ,w_{s},w_{b}$ and the in-plane displacements by u,v and $w_{b},w_{s}$ is measured from middle surface. Furthermore, the shape functions are assumed $\left(z\right)=z-\frac{h}{\pi }\sin\left(\frac{\pi z}{h}\right),g\left(z\right)=1-f^{\prime }\left(z\right)$ . The strain components (Eq. (9)) are computed as follows:

(9) $$\begin{array}{c} \begin{array}{c} \varepsilon_{x}=\frac{\partial u}{\partial \xi }-z\frac{\partial^{2}w_{b}}{\partial x^{2}}-f\left(z\right)\frac{\partial^{2}w_{s}}{\partial x^{2}},\varepsilon_{y}=\frac{\partial v}{\partial y}-z\frac{\partial^{2}w_{b}}{\partial y^{2}}-f\left(z\right)\frac{\partial^{2}w_{s}}{\partial y^{2}},\varepsilon_{z}=\frac{dg\left(z\right)}{dz}\chi , \end{array}\\ \begin{array}{c} \gamma_{xy}=\frac{\partial u}{\partial y}+\frac{\partial v}{\partial x}-2z\frac{\partial^{2}w_{b}}{\partial x\partial y}-2f\left(z\right)\frac{\partial^{2}w_{s}}{\partial x\partial y},\gamma_{xz}=g\left(z\right)\left(\frac{\partial w_{s}}{\partial x}+\frac{\partial \chi }{\partial x}\right),\gamma_{yz}=g\left(z\right)\left(\frac{\partial w_{s}}{\partial y}+\frac{\partial \chi }{\partial y}\right) \end{array} \end{array}$$

The constitutive relations Eq. (10) are obtained for reinforced core as follows:

(10) $$\begin{array}{c} \sigma_{x}=C_{11}\left(\frac{\partial u}{\partial x}-z\frac{\partial^{2}w_{b}}{\partial x^{2}}-f\left(z\right)\frac{\partial^{2}w_{s}}{\partial x^{2}}-\alpha_{eff}T\right)+C_{12}\left(\frac{\partial v}{\partial y}-z\frac{\partial^{2}w_{b}}{\partial y^{2}}-f\left(z\right)\frac{\partial^{2}w_{s}}{\partial y^{2}}-\alpha_{eff}T\right)+C_{13}\left(\frac{dg\left(z\right)}{dz}\chi -\alpha_{eff}T\right)\\ \sigma_{y}=C_{12}\left(\frac{\partial u}{\partial x}-z\frac{\partial^{2}w_{b}}{\partial x^{2}}-f\left(z\right)\frac{\partial^{2}w_{s}}{\partial x^{2}}-\alpha_{eff}T\right)+C_{22}\left(\frac{\partial v}{\partial y}-z\frac{\partial^{2}w_{b}}{\partial y^{2}}-f\left(z\right)\frac{\partial^{2}w_{s}}{\partial y^{2}}-\alpha_{eff}T\right)+C_{23}\left(\frac{dg\left(z\right)}{dz}\chi -\alpha_{eff}T\right)\\ \sigma_{z}=C_{13}\left(\frac{\partial u}{\partial x}-z\frac{\partial^{2}w_{b}}{\partial x^{2}}-f\left(z\right)\frac{\partial^{2}w_{s}}{\partial x^{2}}-\alpha_{eff}T\right)+C_{23}\left(\frac{\partial v}{\partial y}-z\frac{\partial^{2}w_{b}}{\partial y^{2}}-f\left(z\right)\frac{\partial^{2}w_{s}}{\partial y^{2}}-\alpha_{eff}T\right)+C_{33}\left(\frac{dg\left(z\right)}{dz}\chi -\alpha_{eff}T\right)\\ \sigma_{xy}=C_{66}\left(\frac{\partial u}{\partial y}+\frac{\partial v}{\partial x}-2z\frac{\partial^{2}w_{b}}{\partial x\partial y}-2f\left(z\right)\frac{\partial^{2}w_{s}}{\partial x\partial y}\right),\sigma_{xz}=C_{55}\left(g\left(z\right)\left(\frac{\partial w_{s}}{\partial x}+\frac{\partial \chi }{\partial x}\right)\right),\sigma_{yz}=C_{44}\left(g\left(z\right)\left(\frac{\partial w_{s}}{\partial y}+\frac{\partial \chi }{\partial y}\right)\right) \end{array}$$

where, $C_{ij}$ are stiffness coefficients. The constitutive relations Eq. (11) for piezoelectric/piezomagnetic face-sheets are obtained as follows:

(11) $$\begin{array}{c} \sigma_{x}=C_{11}\left(\frac{\partial u}{\partial x}-z\frac{\partial^{2}w_{b}}{\partial x^{2}}-f\left(z\right)\frac{\partial^{2}w_{s}}{\partial x^{2}}-\alpha_{eff}T\right)+C_{12}\left(\frac{\partial v}{\partial y}-z\frac{\partial^{2}w_{b}}{\partial y^{2}}-f\left(z\right)\frac{\partial^{2}w_{s}}{\partial y^{2}}-\alpha_{eff}T\right)+C_{13}\left(\frac{dg\left(z\right)}{dz}\chi -\alpha_{eff}T\right)\;+e_{31}\left(\frac{2}{h}\Psi_{0}+\frac{\pi }{h}\Psi \;\sin\frac{\pi z}{h}\right)+q_{31}\left(\frac{2}{h}\Phi_{0}+\frac{\pi }{h}\Phi \;\sin\frac{\pi z}{h}\right)\\ \sigma_{y}=C_{12}\left(\frac{\partial u}{\partial x}-z\frac{\partial^{2}w_{b}}{\partial x^{2}}-f\left(z\right)\frac{\partial^{2}w_{s}}{\partial x^{2}}-\alpha_{eff}T\right)+C_{22}\left(\frac{\partial v}{\partial y}-z\frac{\partial^{2}w_{b}}{\partial y^{2}}-f\left(z\right)\frac{\partial^{2}w_{s}}{\partial y^{2}}-\alpha_{eff}T\right)+C_{23}\left(\frac{dg\left(z\right)}{dz}\chi -\alpha_{eff}T\right)\;+e_{32}\left(\frac{2}{h}\Psi_{0}+\frac{\pi }{h}\Psi \;\sin\frac{\pi z}{h}\right)+q_{32}\left(\frac{2}{h}\Phi_{0}+\frac{\pi }{h}\Phi \;\sin\frac{\pi z}{h}\right)\\ \sigma_{z}=C_{13}\left(\frac{\partial u}{\partial x}-z\frac{\partial^{2}w_{b}}{\partial x^{2}}-f\left(z\right)\frac{\partial^{2}w_{s}}{\partial x^{2}}-\alpha_{eff}T\right)+C_{32}\left(\frac{\partial v}{\partial y}-z\frac{\partial^{2}w_{b}}{\partial y^{2}}-f\left(z\right)\frac{\partial^{2}w_{s}}{\partial y^{2}}-\alpha_{eff}T\right)+C_{33}\left(\frac{dg\left(z\right)}{dz}\chi -\alpha_{eff}T\right)\;+e_{33}\left(\frac{2}{h}\Psi_{0}+\frac{\pi }{h}\Psi \;\sin\frac{\pi z}{h}\right)+q_{33}\left(\frac{2}{h}\Phi_{0}+\frac{\pi }{h}\Phi \;\sin\frac{\pi z}{h}\right)\\ \sigma_{xy}=C_{66}\left(\frac{\partial u}{\partial y}+\frac{\partial v}{\partial x}-2z\frac{\partial^{2}w_{b}}{\partial x\partial y}-2f\left(z\right)\frac{\partial^{2}w_{s}}{\partial x\partial y}\right),\sigma_{x}=C_{55}\left(g\left(z\right)\left(\frac{\partial w_{s}}{\partial x}+\frac{\partial \chi }{\partial x}\right)\right)-e_{15}\frac{\partial \Psi }{\partial x}\cos\frac{\pi z}{h}-q_{15}\frac{\partial \Phi }{\partial x}\cos\frac{\pi z}{h}\\ \sigma_{yz}=C_{44}\left(g\left(z\right)\left(\frac{\partial w_{s}}{\partial y}+\frac{\partial \chi }{\partial y}\right)\right)-e_{24}\frac{\partial \Psi }{\partial y}\cos\frac{\pi z}{h}-q_{24}\frac{\partial \Phi }{\partial y}\cos\frac{\pi z}{h} \end{array}$$

The multi filed components Eqs. (12), (13) are expressed as:

(12) $$D_{x}=e_{15}\left(g\left(z\right)\left(\frac{\partial w_{s}}{\partial x}+\frac{\partial \chi }{\partial x}\right)\right)+\eta_{11}\frac{\partial \Psi }{\partial x}\cos\frac{\pi z}{h}+d_{11}\frac{\partial \Phi }{\partial x}\cos\frac{\pi z}{h}\;D_{y}=e_{24}\left(g\left(z\right)\left(\frac{\partial w_{s}}{\partial y}+\frac{\partial \chi }{\partial y}\right)\right)+\eta_{22}\frac{\partial \Psi }{\partial y}\cos\frac{\pi z}{h}+d_{22}\frac{\partial \Phi }{\partial y}\cos\frac{\pi z}{h}\;D_{z}=e_{31}\left(\frac{\partial u}{\partial x}-z\frac{\partial^{2}w_{b}}{\partial x^{2}}-f\left(z\right)\frac{\partial^{2}w_{s}}{\partial x^{2}}-\alpha_{eff}T\right)+e_{32}\left(\frac{\partial v}{\partial y}-z\frac{\partial^{2}w_{b}}{\partial y^{2}}-f\left(z\right)\frac{\partial^{2}w_{s}}{\partial y^{2}}-\alpha_{eff}T\right)+e_{33}\left(\frac{dg\left(z\right)}{dz}\chi -\alpha_{eff}T\right)-\eta_{33}\left(\frac{2}{h}\Psi_{0}+\frac{\pi }{h}\Psi \;\sin\frac{\pi z}{h}\right)-d_{33}\left(\frac{2}{h}\Phi_{0}+\frac{\pi }{h}\Phi \;\sin\frac{\pi z}{h}\right)$$

(13) $$B_{x}=q_{15}\left(g\left(z\right)\left(\frac{\partial w_{s}}{\partial x}+\frac{\partial \chi }{\partial x}\right)\right)+d_{11}\frac{\partial \Psi }{\partial x}\cos\frac{\pi z}{h}+\mu_{11}\frac{\partial \Phi }{\partial x}\cos\frac{\pi z}{h}\;B_{y}=q_{24}\left(g\left(z\right)\left(\frac{\partial w_{s}}{\partial y}+\frac{\partial \chi }{\partial y}\right)\right)+d_{22}\frac{\partial \Psi }{\partial y}\cos\frac{\pi z}{h}+\mu_{22}\frac{\partial \Phi }{\partial y}\cos\frac{\pi z}{h}\;B_{z}=q_{31}\left(\frac{\partial u}{\partial x}-z\frac{\partial^{2}w_{b}}{\partial x^{2}}-f\left(z\right)\frac{\partial^{2}w_{s}}{\partial x^{2}}-\alpha_{eff}T\right)+q_{32}\left(\frac{\partial v}{\partial y}-z\frac{\partial^{2}w_{b}}{\partial y^{2}}-f\left(z\right)\frac{\partial^{2}w_{s}}{\partial y^{2}}-\alpha_{eff}T\right)+q_{33}\left(\frac{dg\left(z\right)}{dz}\chi -\alpha_{eff}T\right)-d_{33}\left(\frac{2}{h}\Psi_{0}+\frac{\pi }{h}\Psi \;\sin\frac{\pi z}{h}\right)-\mu_{33}\left(\frac{2}{h}\Phi_{0}+\frac{\pi }{h}\Phi \;\sin\frac{\pi z}{h}\right)$$

The strain energy variation Eq. (14) is expressed as:

(14) $$\delta U=\iint \left(\left[N_{x}\frac{\partial u}{\partial x}-M_{x}\frac{\partial^{2}w_{b}}{\partial x^{2}}-S_{x}\frac{\partial^{2}w_{s}}{\partial x^{2}}\right]+\left[N_{y}\frac{\partial v}{\partial y}-M_{y}\frac{\partial^{2}w_{b}}{\partial y^{2}}-S_{y}\frac{\partial^{2}w_{s}}{\partial y^{2}}\right]+\left[N_{z}\chi \right]+\left[N_{xy}\frac{\partial u}{\partial y}+N_{xy}\frac{\partial v}{\partial x}-2M_{xy}\frac{\partial^{2}w_{b}}{\partial x\partial y}-2S_{xy}\frac{\partial^{2}w_{s}}{\partial x\partial y}\right]+\left[N_{xz}\left(\frac{\partial \delta w_{s}}{\partial x}+\frac{\partial \delta \chi }{\partial x}\right)\right]+\left[N_{yz}\left(\frac{\partial \delta w_{s}}{\partial y}+\frac{\partial \delta \chi }{\partial y}\right)\right]-\bar{D_{x}}\frac{\partial \delta \Psi }{\partial x}-\bar{D_{y}}\frac{\partial \delta \Psi }{\partial y}+\bar{D_{z}}\delta \Psi -\bar{B_{x}}\frac{\partial \delta \Phi }{\partial x}-\bar{B_{y}}\frac{\partial \delta \Phi }{\partial y}+\bar{B_{z}}\delta \Phi \right)dxdy\text{.}$$

By definition of the resultant components Eq. (15) as:

$$\left\{N_{x},M_{x},S_{x}\right\}=\int_{-\frac{h}{2}}^{\frac{h}{2}}\sigma_{x}\left\{1,z,f\left(z\right)\right\}\text{dz},\left\{\bar{D_{x}},\bar{D_{y}},\bar{D_{z}}\right\}=\int_{-\frac{h}{2}}^{\frac{h}{2}}\left\{D_{1}\;\cos\frac{\pi z}{h},D_{2}\;\cos\frac{\pi z}{h},D_{3}\frac{\pi }{h}\sin\frac{\pi z}{h}\right\}\text{dz,}$$

$$\left\{N_{y},M_{y},S_{y}\right\}=\int_{-\frac{h}{2}}^{\frac{h}{2}}\sigma_{y}\left\{1,z,f\left(z\right)\right\}\text{dz},\left\{N_{z}\right\}=\int_{-\frac{h}{2}}^{\frac{h}{2}}\sigma_{z}\frac{dg\left(z\right)}{dz}\text{dz,}$$

(15) $$\left\{N_{xy},M_{xy},S_{xy}\right\}=\int_{-h/2}^{h/2}\tau_{xy}\left\{1,z,f\left(z\right)\right\}\text{dz},\left\{N_{xz},N_{yz}\right\}=\int_{-h/2}^{h/2}\left\{\tau_{xz},\tau_{yz}\right\}g\left(z\right)\text{dz.}$$

New form of strain energy Eq. (16) is computed as follows [[77], [78], [79], [80]]:

(16) $$\delta U=\iint \left(-\frac{\partial N_{x}}{\partial x}\delta u-\frac{\partial N_{xy}}{\partial y}\delta u-\frac{\partial N_{y}}{\partial y}\delta v-\frac{\partial N_{xy}}{\partial x}\delta v-\frac{\partial^{2}M_{x}}{\partial x}\delta w_{b}-\frac{\partial^{2}M_{y}}{\partial y^{2}}\delta w_{b}-2\frac{\partial^{2}M_{xy}}{\partial x\partial y}\delta w_{b}-\frac{\partial^{2}S_{x}}{\partial x^{2}}\delta w_{s}-\frac{\partial^{2}S_{y}}{\partial y^{2}}\delta w_{s}-2\frac{\partial^{2}S_{xy}}{\partial x\partial y}\delta w_{s}-\frac{\partial N_{xz}}{\partial x}\delta w_{s}-\frac{\partial N_{yz}}{\partial y}\delta w_{s}+N_{z}\delta \chi -\frac{\partial N_{xz}}{\partial x}\delta \chi -\frac{\partial N_{yz}}{\partial y}\delta \chi +\frac{\partial \bar{D_{x}}}{\partial x}\delta \Psi +\frac{\partial \bar{D_{y}}}{\partial y}\delta \Psi +\bar{D_{z}}\delta \Psi +\frac{\partial \bar{B_{x}}}{\partial x}\delta \Phi +\frac{\partial \bar{B_{y}}}{\partial y}\delta \Phi +\bar{B_{z}}\delta \Phi \right)dxdy$$

The external works Eq. (17) is defined as follows [[81], [82], [83], [84], [85]]:

(17) $$\delta W=\int_{0}^{L}\left[q{\delta w|}_{z=+h/2}-N_{x}^{T}\frac{\partial^{2}w}{\partial x^{2}}-N_{y}^{T}\frac{\partial^{2}w}{\partial y^{2}}\right]dxdy\text{,}$$

In which $N_{x}^{T},N_{y}^{T}$ are pre-thermal loads along x, y directions and w is total transverse deflection. Using definition of total potential energy as Π=W−U, and minimization δΠ=0 , we will arrive to Eq. (18) [[66], [67], [68], [69], [70], [71]]:

(18) $$\begin{array}{c} \begin{array}{c} \delta u:-\frac{\partial N_{x}}{\partial x}-\frac{\partial N_{xy}}{\partial y}=0,\\ \delta v:-\frac{\partial N_{y}}{\partial y}-\frac{\partial N_{xy}}{\partial x}=0, \end{array}\\ \begin{array}{c} \delta w_{b}:-\frac{\partial^{2}M_{x}}{\partial x^{2}}-\frac{\partial^{2}M_{y}}{\partial y^{2}}-2\frac{\partial^{2}M_{xy}}{\partial x\partial y}=q-N_{x}^{T}\frac{\partial^{2}w_{b}\left(x,y\right)}{\partial x^{2}}-N_{y}^{T}\frac{\partial^{2}w_{b}\left(x,y\right)}{\partial y^{2}},\\ \delta w_{s}:-\frac{\partial^{2}S_{x}}{\partial x^{2}}-\frac{\partial^{2}S_{y}}{\partial y^{2}}-2\frac{\partial^{2}S_{xy}}{\partial x\partial y}-\frac{\partial N_{xz}}{\partial x}-\frac{\partial N_{yz}}{\partial y}=q-N_{x}^{T}\frac{\partial^{2}w_{s}\left(x,y\right)}{\partial x^{2}}-N_{y}^{T}\frac{\partial^{2}w_{s}\left(x,y\right)}{\partial y^{2}},\\ \delta \chi :N_{z}-\frac{\partial N_{xz}}{\partial x}-\frac{\partial N_{yz}}{\partial y}=qg\left(z=+\frac{h}{2}\right)-N_{x}^{T}g\left(0\right)\frac{\partial^{2}\chi \left(x,y\right)}{\partial x^{2}}-N_{y}^{T}g\left(0\right)\frac{\partial^{2}\chi \left(x,y\right)}{\partial y^{2}}\\ \delta \Psi :\frac{\partial \bar{D_{x}}}{\partial x}+\frac{\partial \bar{D_{y}}}{\partial y}+\bar{D_{z}}=0\\ \delta \Psi :\frac{\partial \bar{B_{x}}}{\partial x}+\frac{\partial \bar{B_{y}}}{\partial y}+\bar{B_{z}}=0 \end{array} \end{array}$$

In which [[86], [87], [88], [89], [90]]:

(19) $$\begin{array}{c} \left\{N_{x},N_{y},M_{x},M_{y},S_{x},S_{y},N_{z},D_{z},B_{z}\right\}=\left\{J_{1},J_{4},J_{8},J_{11},J_{15},J_{18},J_{31},R_{25},R_{40}\right\}\frac{\partial u_{0}}{\partial x}-\left\{J_{2},J_{5},J_{9},J_{12},J_{16},J_{19},J_{32},R_{26},R_{41}\right\}\frac{\partial^{2}w_{b}}{\partial x^{2}}-\left\{J_{3},J_{6},J_{10},J_{13},J_{17},J_{20},J_{33},R_{27},R_{42}\right\}\frac{\partial^{2}w_{s}}{\partial y^{2}}+\left\{J_{4},J_{1},J_{11},J_{8},J_{18},J_{15},J_{34},R_{28},R_{43}\right\}\frac{\partial v_{0}}{\partial y}-\left\{J_{5},J_{2},J_{12},J_{9},J_{19},J_{16},J_{35},R_{29},R_{44}\right\}\frac{\partial^{2}w_{b}}{\partial y^{2}}-\left\{J_{6},J_{3},J_{13},J_{10},J_{20},J_{17},J_{36},R_{30},R_{45}\right\}\frac{\partial^{2}w_{s}}{\partial y^{2}}+\left\{J_{7},J_{7},J_{14},J_{14},J_{21},J_{21},J_{37},R_{31},R_{46}\right\}\chi -\left\{J_{1}^{T},J_{1}^{T},J_{2}^{T},J_{2}^{T},J_{3}^{T},J_{3}^{T},J_{4}^{T},J_{5}^{T},J_{6}^{T}\right\}\left(T-T_{0}\right)+\left\{N_{xx}^{\Psi_{0}},N_{yy}^{\Psi_{0}},M_{xx}^{\Psi_{0}},M_{yy}^{\Psi_{0}},S_{xx}^{\Psi_{0}},S_{yy}^{\Psi_{0}},N_{zz}^{\Psi_{0}},-D_{z}^{\Psi_{0}},-B_{z}^{\Psi_{0}}\right\}+\left\{R_{1},R_{7},R_{3},R_{9},R_{5},R_{11},R_{13},-R_{32},-R_{47}\right\}\Psi +\left\{N_{xx}^{\Phi_{0}},N_{yy}^{\Phi_{0}},M_{xx}^{\Phi_{0}},M_{yy}^{\Phi_{0}},S_{xx}^{\Phi_{0}},S_{yy}^{\Phi_{0}},N_{zz}^{\Phi_{0}},{-D}_{z}^{\Phi_{0}},{-B}_{z}^{\Phi_{0}}\right\}+\left\{R_{2},R_{8},R_{4},R_{10},R_{6},R_{12},R_{14},-R_{33},-R_{48}\right\}\Phi \\ \left\{N_{xy},M_{xy},S_{xy}\right\}=\left\{J_{22},J_{25},J_{28}\right\}\frac{\partial u_{0}}{\partial y}+\left\{J_{22},J_{25},J_{28}\right\}\frac{\partial v_{0}}{\partial x}-2\left\{J_{23},J_{26},J_{29}\right\}\frac{\partial^{2}w_{b}}{\partial x\partial y}-2\left\{J_{24},J_{27},J_{30}\right\}\frac{\partial^{2}w_{s}}{\partial x\partial y}\\ \begin{array}{c} \left\{N_{\xi Z},N_{\zeta Z}\right\}=\left\{J_{38},J_{39}\right\}\left(\frac{\partial w_{s}}{\partial x}+\frac{\partial \chi }{\partial x}\right)-\left\{R_{15},R_{17}\right\}\frac{\partial \Psi }{\partial x}-\left\{R_{16},R_{18}\right\}\frac{\partial \Phi }{\partial x}\\ \left\{D_{\xi },B_{\xi }\right\}=\left\{R_{19},R_{34}\right\}\left(\frac{\partial w_{s}}{\partial x}+\frac{\partial \chi }{\partial x}\right)+\left\{R_{21},R_{36}\right\}\frac{\partial \Psi }{\partial x}+\left\{R_{22},R_{37}\right\}\frac{\partial \Phi }{\partial x}\\ \left\{D_{\zeta },B_{\zeta }\right\}=\left\{R_{20},R_{35}\right\}\left(\frac{\partial w_{s}}{\partial x}+\frac{\partial \chi }{\partial x}\right)+\left\{R_{23},R_{38}\right\}\frac{\partial \Psi }{\partial y}+\left\{R_{24},R_{39}\right\}\frac{\partial \Phi }{\partial y} \end{array} \end{array}$$

In which the integration constants in Eq. (19) are presented in Appendix A. Finally, the governing equations are derived as Eq. (20):

(20) $$\begin{array}{c} \begin{array}{c} \delta u:J_{1}\frac{\partial^{2}u_{0}}{\partial x^{2}}+J_{22}\frac{\partial^{2}u_{0}}{\partial y^{2}}+\left(J_{4}+J_{22}\right)\frac{\partial^{2}v_{0}}{\partial x\partial y}-J_{2}\frac{\partial^{3}w_{b}}{\partial x^{3}}-\left(2J_{23}+J_{5}\right)\frac{\partial^{3}w_{b}}{\partial x\partial y^{2}}-J_{3}\frac{\partial^{3}w_{s}}{\partial x^{3}}-\left(J_{6}+2J_{24}\right)\frac{\partial^{3}w_{s}}{\partial x\partial y^{2}}\;+J_{7}\frac{\partial \chi }{\partial x}+R_{1}\frac{\partial \Psi }{\partial x}+R_{2}\frac{\partial \Phi }{\partial x}+\frac{\partial N_{xx}^{\Psi_{0}}}{\partial x}+\frac{\partial N_{xx}^{\Phi_{0}}}{\partial x}-J_{1}^{T}\frac{\partial \left(T-T_{0}\right)}{\partial x}=0,\\ \delta v:\left(J_{4}+J_{22}\right)\frac{\partial^{2}u_{0}}{\partial x\partial y}+J_{22}\frac{\partial^{2}v_{0}}{\partial x^{2}}+J_{1}\frac{\partial^{2}v_{0}}{\partial y^{2}}-J_{2}\frac{\partial^{3}w_{b}}{\partial y^{3}}-\left(J_{5}+2J_{23}\right)\frac{\partial^{3}w_{b}}{\partial x^{2}\partial y}-J_{3}\frac{\partial^{3}w_{s}}{\partial y^{3}}-\left(J_{6}+2J_{24}\right)\frac{\partial^{3}w_{s}}{\partial x^{2}\partial y}\;+J_{7}\frac{\partial \chi }{\partial y}+R_{7}\frac{\partial \Psi }{\partial y}+R_{8}\frac{\partial \Phi }{\partial y}-J_{1}^{T}\frac{\partial \left(T-T_{0}\right)}{\partial y}+\frac{\partial N_{yy}^{\Psi_{0}}}{\partial y}+\frac{\partial N_{yy}^{\Phi_{0}}}{\partial y}=0, \end{array}\\ \begin{array}{c} \delta w_{b}:J_{8}\frac{\partial^{3}u_{0}}{\partial x^{3}}+\left(J_{11}+2J_{25}\right)\frac{\partial^{3}u_{0}}{\partial x\partial y^{2}}+J_{8}\frac{\partial^{3}v_{0}}{\partial y^{3}}+\left(J_{11}+2J_{25}\right)\frac{\partial^{3}v_{0}}{\partial x^{2}\partial y}-{Ρ}_{9}\frac{\partial^{4}w_{b}}{\partial x^{4}}-J_{9}\frac{\partial^{4}w_{b}}{\partial y^{4}}-\left(J_{12}+4J_{26}+J_{12}\right)\frac{\partial^{4}w_{b}}{\partial x^{2}\partial y^{2}}-J_{10}\frac{\partial^{4}w_{s}}{\partial {yx}^{4}}-J_{10}\frac{\partial^{4}w_{s}}{\partial y^{4}}-\left(J_{13}+J_{13}+4J_{27}\right)\frac{\partial^{4}w_{s}}{\partial x^{2}\partial y^{2}}+J_{14}\frac{\partial^{2}\chi }{\partial x^{2}}+J_{14}\frac{\partial^{2}\chi }{\partial y^{2}}+R_{3}\frac{\partial^{2}\Psi }{\partial x^{2}}+R_{9}\frac{\partial^{2}\Psi }{\partial y^{2}}+R_{4}\frac{\partial^{2}\Phi }{\partial x^{2}}+R_{10}\frac{\partial^{2}\Phi }{\partial y^{2}}-J_{2}^{T}\frac{\partial^{2}\left(T-T_{0}\right)}{\partial x^{2}}+\frac{\partial^{2}M_{xx}^{\Psi_{0}}}{\partial x^{2}}+\frac{\partial^{2}M_{xx}^{\Phi_{0}}}{\partial x^{2}}-J_{2}^{T}\frac{\partial^{2}\left(T-T_{0}\right)}{\partial y^{2}}+\frac{\partial^{2}M_{yy}^{\Psi_{0}}}{\partial y^{2}}+\frac{\partial^{2}M_{yy}^{\Phi_{0}}}{\partial y^{2}}=-q+F_{f}+N_{x}^{T}\frac{\partial^{2}w_{b}\left(x,y\right)}{\partial x^{2}}+N_{y}^{T}\frac{\partial^{2}w_{b}\left(x,y\right)}{\partial y^{2}},\\ \delta w_{s}:J_{15}\frac{\partial^{3}u_{0}}{\partial x^{3}}+\left(2J_{28}+J_{18}\right)\frac{\partial^{3}u_{0}}{\partial x\partial y^{2}}+J_{15}\frac{\partial^{3}v_{0}}{\partial y^{3}}+\left(2J_{28}+J_{18}\right)\frac{\partial^{3}v_{0}}{\partial x^{2}\partial y}-J_{16}\frac{\partial^{4}w_{b}}{\partial x^{4}}-J_{16}\frac{\partial^{4}w_{b}}{\partial y^{4}}-\left(J_{19}+4J_{29}+J_{19}\right)\frac{\partial^{4}w_{b}}{\partial x^{2}\partial y^{2}}-J_{17}\frac{\partial^{4}w_{s}}{\partial x^{4}}-J_{17}\frac{\partial^{4}w_{s}}{\partial y^{4}}-\left(J_{20}+J_{20}+4J_{30}\right)\frac{\partial^{4}w_{s}}{\partial x^{2}\partial y^{2}}+J_{38}\frac{\partial^{2}w_{s}}{\partial x^{2}}+J_{39}\frac{\partial^{2}w_{s}}{\partial \zeta^{2}}+\left(J_{38}+J_{21}\right)\frac{\partial^{2}\chi }{\partial x^{2}}+\left(J_{39}+J_{21}\right)\frac{\partial^{2}\chi }{\partial y^{2}}\;+\left(R_{5}-R_{15}\right)\frac{\partial^{2}\Psi }{\partial x^{2}}+\left(R_{11}-R_{17}\right)\frac{\partial^{2}\Psi }{\partial y^{2}}+\left(R_{6}-R_{16}\right)\frac{\partial^{2}\Phi }{\partial x^{2}}+\left(R_{12}-R_{18}\right)\frac{\partial^{2}\Phi }{\partial y^{2}}\;+\frac{\partial^{2}S_{xx}^{\Psi_{0}}}{\partial x^{2}}+\frac{\partial^{2}S_{xx}^{\Phi_{0}}}{\partial x^{2}}+\frac{\partial^{2}S_{yy}^{\Phi_{0}}}{\partial y^{2}}-J_{3}^{T}\frac{\partial^{2}\left(T-T_{0}\right)}{\partial x^{2}}-J_{3}^{T}\frac{\partial^{2}\left(T-T_{0}\right)}{\partial y^{2}}+\frac{\partial^{2}S_{yy}^{\Psi_{0}}}{\partial y^{2}}=-q+F_{f}+N_{x}^{T}\frac{\partial^{2}w_{s}\left(x,y\right)}{\partial x^{2}}+N_{y}^{T}\frac{\partial^{2}w_{s}\left(x,y\right)}{\partial y^{2}},\\ \delta \chi :-J_{31}\frac{\partial u_{0}}{\partial x}-J_{34}\frac{\partial v_{0}}{\partial y}+J_{32}\frac{\partial^{2}w_{b}}{\partial x^{2}}+J_{35}\frac{\partial^{2}w_{b}}{\partial y^{2}}+\left(J_{33}+J_{38}\right)\frac{\partial^{2}w_{s}}{\partial x^{2}}+\left(J_{36}+J_{39}\right)\frac{\partial^{2}w_{s}}{\partial y^{2}}+J_{38}\frac{\partial^{2}\chi }{\partial x^{2}}+J_{39}\frac{\partial^{2}\chi }{\partial y^{2}}-J_{37}\chi -R_{15}\frac{\partial^{2}\Psi }{\partial x^{2}}-R_{17}\frac{\partial^{2}\Psi }{\partial y^{2}}-R_{13}\Psi -R_{16}\frac{\partial^{2}\Phi }{\partial x^{2}}-R_{18}\frac{\partial^{2}\Phi }{\partial y^{2}}-R_{14}\Phi +J_{4}^{T}\left(T-T_{0}\right)-N_{zz}^{\Psi_{0}}-N_{zz}^{\Phi_{0}}=-qg\left(z=+\frac{h}{2}\right)+F_{f}g\left(z=-\frac{h}{2}\right)+N_{x}^{T}g\left(0\right)\frac{\partial^{2}\chi \left(x,y\right)}{\partial x^{2}}+N_{y}^{T}g\left(0\right)\frac{\partial^{2}\chi \left(x,y\right)}{\partial y^{2}}\\ \delta \Psi :R_{25}\frac{\partial u_{0}}{\partial x}+R_{28}\frac{\partial v_{0}}{\partial y}-R_{26}\frac{\partial^{2}w_{b}}{\partial x^{2}}-R_{29}\frac{\partial^{2}w_{b}}{\partial y^{2}}+\left(R_{19}-R_{27}\right)\frac{\partial^{2}w_{s}}{\partial x^{2}}+\left(R_{20}-R_{30}\right)\frac{\partial^{2}w_{s}}{\partial y^{2}}+R_{19}\frac{\partial^{2}\chi }{\partial x^{2}}+R_{20}\frac{\partial^{2}\chi }{\partial y^{2}}+R_{31}\chi \;+R_{21}\frac{\partial^{2}\Psi }{\partial x^{2}}+R_{23}\frac{\partial^{2}\Psi }{\partial y^{2}}-R_{32}\Psi +R_{22}\frac{\partial^{2}\Phi }{\partial x^{2}}+R_{24}\frac{\partial^{2}\Phi }{\partial y^{2}}-R_{33}\Phi -D_{z}^{\Psi_{0}}{-D}_{z}^{\Phi_{0}}-J_{5}^{T}\left(T-T_{0}\right)=0\\ \delta \Phi :R_{40}\frac{\partial u_{0}}{\partial x}+R_{43}\frac{\partial v_{0}}{\partial y}-R_{41}\frac{\partial^{2}w_{b}}{\partial x^{2}}-R_{44}\frac{\partial^{2}w_{b}}{\partial y^{2}}+\left(R_{34}-R_{42}\right)\frac{\partial^{2}w_{s}}{\partial x^{2}}+\left(R_{35}-R_{45}\right)\frac{\partial^{2}w_{s}}{\partial y^{2}}+R_{34}\frac{\partial^{2}\chi }{\partial x^{2}}+R_{35}\frac{\partial^{2}\chi }{\partial y^{2}}+R_{46}\chi \;+R_{36}\frac{\partial^{2}\Psi }{\partial x^{2}}+R_{38}\frac{\partial^{2}\Psi }{\partial y^{2}}-R_{47}\Psi +R_{37}\frac{\partial^{2}\Phi }{\partial x^{2}}+R_{39}\frac{\partial^{2}\Phi }{\partial y^{2}}-R_{48}\Phi -B_{z}^{\Psi_{0}}{-B}_{z}^{\Phi_{0}}-J_{6}^{T}\left(T-T_{0}\right)=0 \end{array} \end{array}$$

## 3 Analytical solution

Analytical solution for multi-field analysis of a thickness-stretchable sandwich plate is demonstrated in this section. The unknown functions are assumed using trigonometric functions for simply supported boundary conditions using Eq. (21) as follows:

(21) $$\left\{\begin{array}{c} u_{0}\\ v_{0}\\ w_{b}\\ w_{s}\\ \chi \\ \Psi \\ \Phi \end{array}\right\}=\sum_{m=1}^{\infty }\sum_{n=1}^{\infty }\left\{\begin{array}{c} U_{mn}\;\cos\frac{m\pi x}{a}\sin\frac{n\pi y}{b}\\ V_{mn}\;\sin\frac{m\pi x}{a}\cos\frac{n\pi y}{b}\\ W_{mn}^{b}\;\sin\frac{m\pi x}{a}\sin\frac{n\pi y}{b}\\ W_{mn}^{s}\;\sin\frac{m\pi x}{a}\sin\frac{n\pi y}{b}\\ X_{mn}\;\sin\frac{m\pi x}{a}\sin\frac{n\pi y}{b}\\ \Psi_{mn}\;\sin\frac{m\pi x}{a}\sin\frac{n\pi y}{b}\\ \Phi_{mn}\;\sin\frac{m\pi x}{a}\sin\frac{n\pi y}{b} \end{array}\right\}$$

where $U_{mn},V_{mn},W_{mn}^{b},W_{mn}^{s},X_{mn},\Psi_{mn},\Phi_{mn}$ are unknown displacements, electric and magnetic potentials. Final equation [K]{X} = {F} is obtained through substitution of solution from Eq. (21) into governing equation Eq. 20 [91].

## 4 Results and discussion

Deformation, strain and stress parametric analyses of a composite plate composed of cooper matrix reinforced with graphene origami are explored in this section. To present a justified analysis, a verification study is explored before parametric analysis.

Two Tables are prepared here to compare present results with those results available in the previous references. Table 1 lists changes of the dimensionless transverse deflection with changes of length to thickness ratio L/h based on valid reports [[41], [42], [43]]. Readers can approve agreement of the present results through comparison with available results. Furthermore, to investigate effect of zero out of plane normal strain (thickness stretched model), Table 2 is organized to present dimensionless shear and normal stresses and deflection based on present formulation and available works [44,45].

**Table 1:** Changes of the dimensionless transverse deflection with changes of length to thickness ratio L/h based on valid reports [[41], [42], [43]].

L/h	Ying et al. [41]	Chen et al. [42]	H. Ait Atmane et al. [43]	Present
5	3.06373	3.04799	3.04842	3.04823
15	3.13227	3.13025	3.13093	3.13022
120	3.14145	3.14143	3.14214	3.14189

**Table 2:** Changes of dimensionless shear and normal stresses and deflection based on present formulation and available works [44,45].

	Reference [44] ($\varepsilon_{z}\neq 0$)	Reference [44] ($\varepsilon_{z}=0$)	Reference [45]	Present
$\bar{w}$	0.4616338	0.4802619	0.4616336	0.461603
$\bar{\sigma_{x}}$	−2.2868928	−2.0796748	−2.2868927	−2.286876
$\bar{\tau_{xz}}$	0.7624377	0.5740629	0.7624379	0.762402

After the comparative study, the complete numerical results are presented. The material properties of Cu matrix and graphene origami as assumed as [38]:

$$E_{Cu}=65.79GPa,E_{Gr}=929.57GPa,\vartheta_{Cu}=0.387,\vartheta_{Gr}=0.22\text{,}$$

$$\alpha_{Cu}=16.51\times 10^{-6}\frac{1}{K^{O}},\vartheta_{Gr}=-3.98\times 10^{-6}\frac{1}{K^{O}},l_{Gr}=83.76\times 10^{-10}m,t_{Gr}=3.4\times 10^{-10}m\text{,}$$

The full numerical results are presented in this section. The numerical results are included radial and axial displacements, axial, radial, circumferential and shear stress and strain components.

Table 3 lists variation in dimensionless bending deflection $\bar{w_{b}}$ in terms of initial magnetic potential $\Phi_{0}$ for various initial electric potentials $\Psi_{0}$. An increase in $\bar{w_{b}}$ is observed with an increase in $\Psi_{0}$ and $\Phi_{0}$. The results presented in Table 3, Table 4, Table 5, Table 6, Table 7 are derived for $T=350^{O}K$.

**Table 3:** The variation in $\bar{w_{b}}$ in terms of $\Phi_{0}$ for various $\Psi_{0}$.

$\Phi_{0}$	$\Psi_{0}=0$	$\Psi_{0}=10$	$\Psi_{0}=20$	$\Psi_{0}=30$	$\Psi_{0}=40$	$\Psi_{0}=50$
0	−0.052384	−0.052420	−0.052456	−0.052492	−0.052528	−0.052564
0.1	−0.052398	−0.052434	−0.052470	−0.052505	−0.052541	−0.052577
0.2	−0.052411	−0.052447	−0.052483	−0.052519	−0.052555	−0.052591
0.3	−0.052425	−0.052461	−0.052497	−0.052532	−0.052568	−0.052604
0.4	−0.052438	−0.052474	−0.052510	−0.052546	−0.052582	−0.052618
0.5	−0.052452	−0.052488	−0.052524	−0.052559	−0.052595	−0.052631
0.6	−0.052465	−0.052501	−0.052537	−0.052573	−0.052609	−0.052645
0.7	−0.052479	−0.052515	−0.052551	−0.052586	−0.052622	−0.052658
0.8	−0.052492	−0.052528	−0.052564	−0.052600	−0.052636	−0.052672
0.9	−0.052506	−0.052542	−0.052578	−0.052613	−0.052649	−0.052685
1	−0.052519	−0.052555	−0.052591	−0.052627	−0.052663	−0.052699

**Table 4:** The variation in dimensionless shear deflection $\bar{w_{s}}$ in terms of initial magnetic potential $\Phi_{0}$ for various initial electric potentials $\Psi_{0}$.

$\Phi_{0}$	$\Psi_{0}=0$	$\Psi_{0}=10$	$\Psi_{0}=20$	$\Psi_{0}=30$	$\Psi_{0}=40$	$\Psi_{0}=50$
0	9.9830E-07	9.9898E-07	9.9966E-07	1.0003E-06	1.0010E-06	1.0017E-06
0.1	9.9855E-07	9.9924E-07	9.9992E-07	1.0006E-06	1.0013E-06	1.0020E-06
0.2	9.9881E-07	9.9949E-07	1.0002E-06	1.0009E-06	1.0015E-06	1.0022E-06
0.3	9.9907E-07	9.9975E-07	1.0004E-06	1.0011E-06	1.0018E-06	1.0025E-06
0.4	9.9932E-07	1.0000E-06	1.0007E-06	1.0014E-06	1.0021E-06	1.0027E-06
0.5	9.9958E-07	1.0003E-06	1.0009E-06	1.0016E-06	1.0023E-06	1.0030E-06
0.6	9.9984E-07	1.0005E-06	1.0012E-06	1.0019E-06	1.0026E-06	1.0033E-06
0.7	1.0001E-06	1.0008E-06	1.0015E-06	1.0021E-06	1.0028E-06	1.0035E-06
0.8	1.0004E-06	1.0010E-06	1.0017E-06	1.0024E-06	1.0031E-06	1.0038E-06
0.9	1.0006E-06	1.0013E-06	1.0020E-06	1.0027E-06	1.0033E-06	1.0040E-06
1	1.0009E-06	1.0015E-06	1.0022E-06	1.0029E-06	1.0036E-06	1.0043E-06

**Table 5:** The variation in dimensionless stretching deflection $\bar{\chi }$ in terms of initial magnetic potential $\Phi_{0}$ for various initial electric potentials $\Psi_{0}$.

$\Phi_{0}$	$\Psi_{0}=0$	$\Psi_{0}=10$	$\Psi_{0}=20$	$\Psi_{0}=30$	$\Psi_{0}=40$	$\Psi_{0}=50$
0	7.1586E-05	7.1635E-05	7.1684E-05	7.1733E-05	7.1782E-05	7.1831E-05
0.1	7.1605E-05	7.1654E-05	7.1703E-05	7.1752E-05	7.1801E-05	7.1850E-05
0.2	7.1623E-05	7.1672E-05	7.1721E-05	7.1770E-05	7.1819E-05	7.1868E-05
0.3	7.1642E-05	7.1691E-05	7.1740E-05	7.1789E-05	7.1838E-05	7.1887E-05
0.4	7.1660E-05	7.1709E-05	7.1758E-05	7.1807E-05	7.1856E-05	7.1905E-05
0.5	7.1679E-05	7.1728E-05	7.1777E-05	7.1826E-05	7.1875E-05	7.1924E-05
0.6	7.1697E-05	7.1746E-05	7.1795E-05	7.1844E-05	7.1893E-05	7.1942E-05
0.7	7.1715E-05	7.1764E-05	7.1813E-05	7.1862E-05	7.1911E-05	7.1960E-05
0.8	7.1734E-05	7.1783E-05	7.1832E-05	7.1881E-05	7.1930E-05	7.1979E-05
0.9	7.1752E-05	7.1801E-05	7.1850E-05	7.1899E-05	7.1948E-05	7.1997E-05
1	7.1771E-05	7.1820E-05	7.1869E-05	7.1918E-05	7.1967E-05	7.2016E-05

**Table 6:** The variation in Ψ in terms of $\Phi_{0}$ for various $\Psi_{0}$.

$\Phi_{0}$	$\Psi_{0}=0$	$\Psi_{0}=10$	$\Psi_{0}=20$	$\Psi_{0}=30$	$\Psi_{0}=40$	$\Psi_{0}=50$
0	0.0200818	0.0200956	0.0201093	0.0201231	0.0201368	0.0201506
0.1	0.0200870	0.0201008	0.0201145	0.0201283	0.0201420	0.0201557
0.2	0.0200922	0.0201059	0.0201197	0.0201334	0.0201472	0.0201609
0.3	0.0200974	0.0201111	0.0201249	0.0201386	0.0201523	0.0201661
0.4	0.0201025	0.0201163	0.0201300	0.0201438	0.0201575	0.0201713
0.5	0.0201077	0.0201215	0.0201352	0.0201489	0.0201627	0.0201764
0.6	0.0201129	0.0201266	0.0201404	0.0201541	0.0201679	0.0201816
0.7	0.0201180	0.0201318	0.0201455	0.0201593	0.0201730	0.0201868
0.8	0.0201232	0.0201370	0.0201507	0.0201645	0.0201782	0.0201920
0.9	0.0201284	0.0201421	0.0201559	0.0201696	0.0201834	0.0201971
1	0.0201336	0.0201473	0.0201611	0.0201748	0.0201886	0.0202023

**Table 7:** The variation in Φ in terms of $\Phi_{0}$ for various $\Psi_{0}$.

$\Phi_{0}$	$\Psi_{0}=0$	$\Psi_{0}=10$	$\Psi_{0}=20$	$\Psi_{0}=30$	$\Psi_{0}=40$	$\Psi_{0}=50$
0	−9.77589E-05	−9.78259E-05	−9.78928E-05	−9.79597E-05	−9.80266E-05	−9.80935E-05
0.1	−9.77841E-05	−9.78510E-05	−9.79179E-05	−9.79849E-05	−9.80518E-05	−9.81187E-05
0.2	−9.78093E-05	−9.78762E-05	−9.79431E-05	−9.80100E-05	−9.80770E-05	−9.81439E-05
0.3	−9.78345E-05	−9.79014E-05	−9.79683E-05	−9.80352E-05	−9.81021E-05	−9.81691E-05
0.4	−9.78596E-05	−9.79266E-05	−9.79935E-05	−9.80604E-05	−9.81273E-05	−9.81942E-05
0.5	−9.78848E-05	−9.79517E-05	−9.80187E-05	−9.80856E-05	−9.81525E-05	−9.82194E-05
0.6	−9.79100E-05	−9.79769E-05	−9.80438E-05	−9.81108E-05	−9.81777E-05	−9.82446E-05
0.7	−9.79352E-05	−9.80021E-05	−9.80690E-05	−9.81359E-05	−9.82029E-05	−9.82698E-05
0.8	−9.79604E-05	−9.80273E-05	−9.80942E-05	−9.81611E-05	−9.82280E-05	−9.82950E-05
0.9	−9.79855E-05	−9.80525E-05	−9.81194E-05	−9.81863E-05	−9.82532E-05	−9.83201E-05
1	−9.80107E-05	−9.80776E-05	−9.81445E-05	−9.82115E-05	−9.82784E-05	−9.83453E-05

Table 4 lists variation in dimensionless shear deflection $\bar{w_{s}}$ in terms of $\Phi_{0}$ for various $\Psi_{0}$. The results show an enhancement in $\bar{w_{s}}$ through an enhancement in $\Psi_{0}$ and $\Phi_{0}$.

Table 5 lists changes of stretching $\bar{\chi }$ in terms of $\Phi_{0}$ for various $\Psi_{0}$. An increase in stretching $\bar{\chi }$ is observed with an increase in $\Psi_{0}$ and $\Phi_{0}$.

Impact of initial multifield loading is studied on the maximum electromagnetic loads. Table 6, Table 7 lists variation in maximum electric Ψ and magnetic Φ potentials in terms of $\Phi_{0}$ for various $\Psi_{0}$, respectively. The results show an increase in both maximum electric and magnetic potentials with an increase in $\Psi_{0}$ and $\Phi_{0}$.

**Fig. 2:**
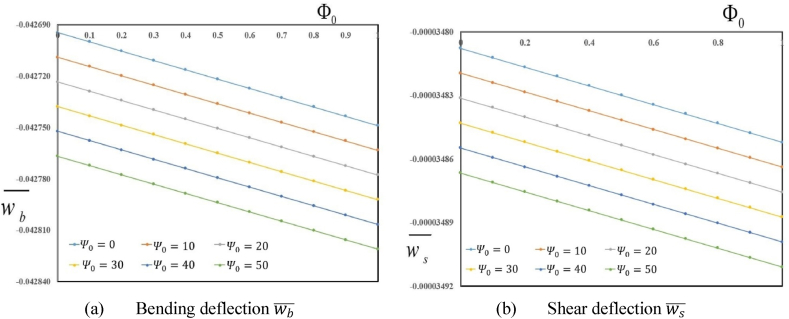
Variation in bending components $\bar{w_{b}}$ and $\bar{w_{s}}$ in terms of $\Phi_{0}$ for various $\Psi_{0}=50$.

Fig. 2 (a, b) shows variation in bending components $\bar{w_{b}}$ and $\bar{w_{s}}$ deflections in terms of initial magnetic potential $\Phi_{0}$ for various initial electric potentials $\Psi_{0}$. The results presented in the Figures are derived for $T=400^{O}K$. The results show that the value of shear deflection is less than bending one. It is concluded that this ratio is obtained for thin plate and for the thicker plate, this value becomes very important and comparable. The results show that the bending and shear deflections are significantly increased with an increase in electrical and magnetic loads.

**Fig. 3:**
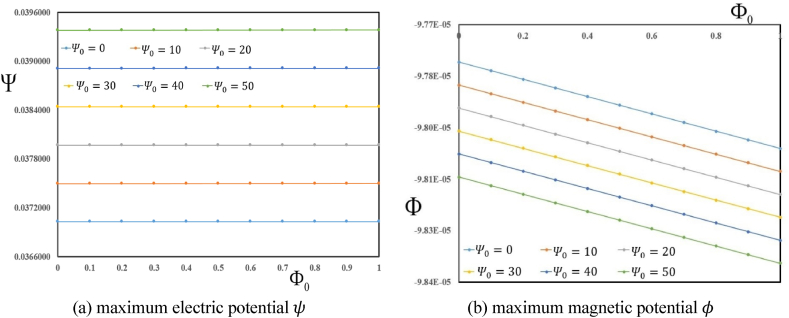
Variation in maximum electric ψ and magnetic φ potentials in terms of initial magnetic potential $\Phi_{0}$ for various initial electric potentials $\Psi_{0}$.

Shown in Fig. 3 (a, b) are variation in maximum electric ψ and magnetic φ potentials in terms of $\Phi_{0}$ for various $\Psi_{0}$. The results show that initial magnetic potential $\Phi_{0}$ has no significant effect on the variation of maximum electric potential ψ. Furthermore, the maximum magnetic potential is increased with an increase in $\Phi_{0}$ and $\Psi_{0}$.

**Fig. 4:**
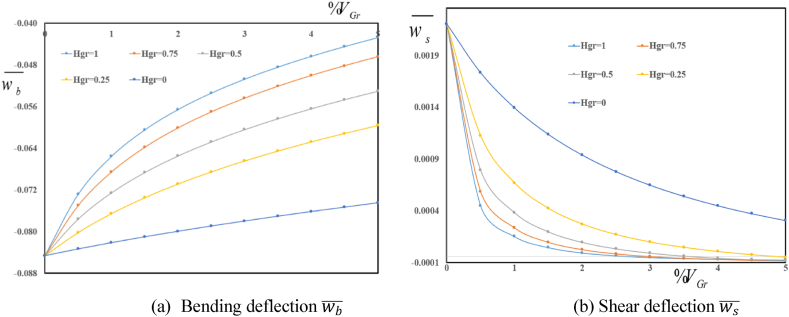
Variation in dimensionless bending $\bar{w_{b}}$ and shear $\bar{w_{s}}$ deflections in terms of volume fractions $V_{Gr}$ for various folding degree $H_{Gr}$.

Depicted in Fig. 4 (a, b) are variation in deflection components $\bar{w_{b}}$ and $\bar{w_{s}}$ in terms of folding degree $H_{Gr}$ for various volume fractions $V_{Gr}$. A decrease in both $\bar{w_{b}}$ and $\bar{w_{s}}$ is observed with an increase in volume fraction of graphene origami. Furthermore, an increase in folding degree $H_{Gr}$ leads to an increase in absolute value of $\bar{w_{b}}$ and $\bar{w_{s}}$. One can conclude that an increase in folding degree leads to a decrease in structural stiffness of reinforced sandwich plate.

**Fig. 5:**
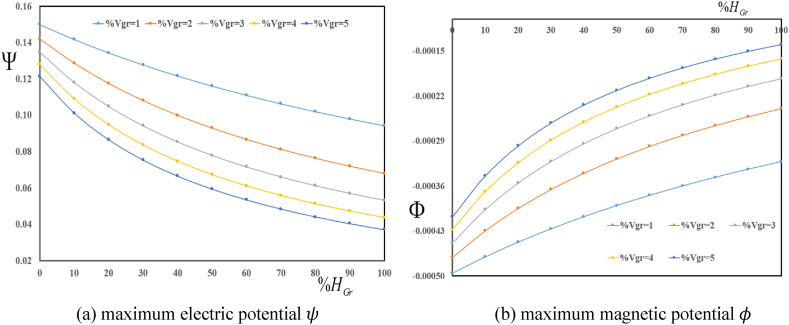
Variation in maximum electric ψ and magnetic φ potentials in terms of volume fractions $V_{Gr}$ for various folding degree $H_{Gr}$.

Fig. 5 (a, b) investigates changes of maximum multifield loads ψ and φ in terms of volume fractions $V_{Gr}$ for various folding degree $H_{Gr}$. The results show a decrease in maximum electric and magnetic potentials with an increase in folding degree $H_{Gr}$. Furthermore, an increase in volume fractions $V_{Gr}$ leads to a decrease in ψ and φ.

**Fig. 6:**
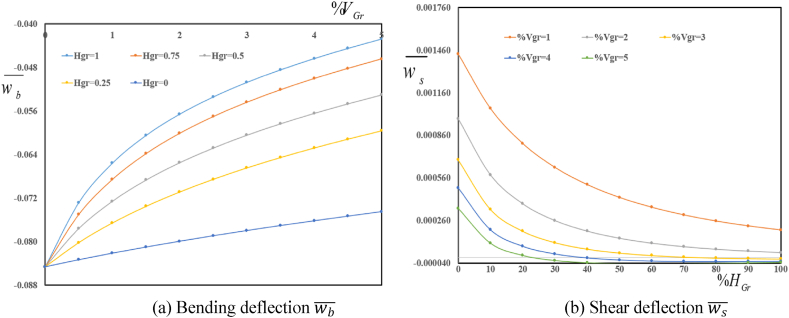
Variation in $\bar{w_{b}}$ and $\bar{w_{s}}$ in terms of folding degree $H_{Gr}$ for various volume fractions $V_{Gr}$.

Fig. 6 (a, b) variation in dimensionless bending $\bar{w_{b}}$ and shear $\bar{w_{s}}$ deflections in terms of folding degree $H_{Gr}$ for various volume fractions $V_{Gr}$. A decrease in both bending and shear deflections is observed with an increase in volume fractions $V_{Gr}$ and folding degree $H_{Gr}$.

**Fig. 7:**
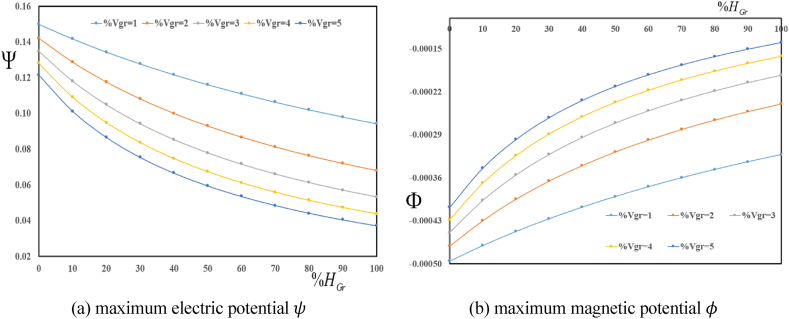
Variation in maximum electric ψ and magnetic φ potentials in terms of folding degree $H_{Gr}$ for various volume fractions $V_{Gr}$.

Fig. 7 (a, b) explores effect of volume characteristic and hydrogenation on the maximum multifield loading inside the plate. These figures show variation in ψ and φ in terms of folding degree $H_{Gr}$ for various volume fractions $V_{Gr}$. The results show a decrease in maximum ψ and φ with an increase in folding degree $H_{Gr}$. Furthermore, an increase in volume fractions $V_{Gr}$ leads to a decrease in ψ and φ.

**Fig. 8:**
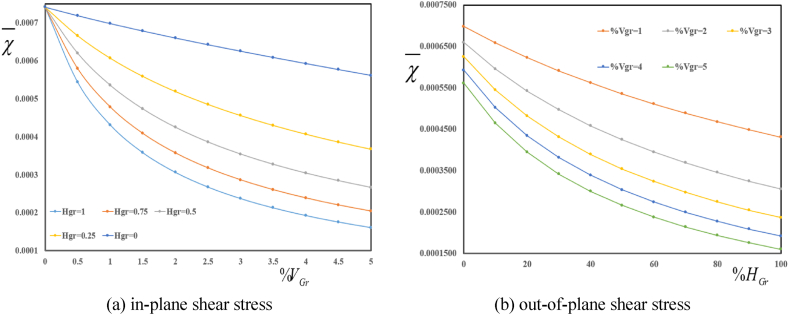
Variation in dimensionless thickness stretching displacement $\bar{\chi }$ in terms of volume fractions $V_{Gr}$ for various folding degree $H_{Gr}$.

Presented in Fig. 8 (a, b) is variation in dimensionless thickness stretching displacement $\bar{\chi }$ in terms of volume fractions $V_{Gr}$ for various folding degree $H_{Gr}$. The results show a decrease in maximum ψ and φ with an increase in folding degree $H_{Gr}$. Furthermore, an increase in volume fractions $V_{Gr}$ leads to a decrease in maximum ψ and φ.

The effect of thermal loading on the deformation, strain and stress components is investigated in the following tables.

Table 8 lists variation in with changes of thermal loading. It is observed an increase in all bending, shear and stretching functions, maximum electric and magnetic potentials with an increase in thermal loading.

**Table 8:** Variation in bending, shear, stretching functions, maximum electric and magnetic potentials with changes of thermal loading

T	$W_{b}$	$W_{s}$	χ	Ψ	Φ
350	−9.57391E-06	5.40756E-08	3.61364E-08	0.069471607	−0.00026114
355	−9.58413E-06	6.14643E-08	4.05054E-08	0.076853566	−0.00028253
360	−9.60053E-06	6.95605E-08	4.49549E-08	0.084376115	−0.00030435
365	−9.6218E-06	7.84407E-08	4.94874E-08	0.092042313	−0.00032662
370	−9.64703E-06	8.81936E-08	5.41053E-08	0.099855455	−0.00034934
375	−9.67555E-06	9.89213E-08	5.88112E-08	0.107819037	−0.00037251
380	−9.70687E-06	1.10742E-07	6.36076E-08	0.115936742	−0.00039614
385	−9.74064E-06	1.23795E-07	6.84969E-08	0.124212425	−0.00042024
390	−9.77657E-06	1.38241E-07	7.34819E-08	0.132650114	−0.00044481
395	−9.81445E-06	1.54271E-07	7.85653E-08	0.141254005	−0.00046987
400	−9.85413E-06	1.72112E-07	8.37499E-08	0.150028459	−0.00049543

Listed in Table 9, Table 10 are variation in all strain and stress components with an increase in thermal loading, respectively. An increase in all stress and strain components is observed with an increase in thermal loading.

**Table 9:** Variation in all normal and shear stress components with changes of thermal loading

T	$\sigma_{x}$	$\sigma_{y}$	$\sigma_{z}$	$\sigma_{xy}$	$\sigma_{xz}$	$\sigma_{yz}$
350	−2.74E+06	−2.74E+06	−3.16E+06	2.67E+04	6.69E+03	6.69E+03
355	−3.11E+06	−3.11E+06	−3.59E+06	2.67E+04	7.54E+03	7.54E+03
360	−3.51E+06	−3.51E+06	−4.03E+06	2.66E+04	8.45E+03	8.45E+03
365	−3.93E+06	−3.93E+06	−4.51E+06	2.66E+04	9.41E+03	9.41E+03
370	−4.38E+06	−4.38E+06	−5.01E+06	2.66E+04	1.04E+04	1.04E+04
375	−4.86E+06	−4.86E+06	−5.55E+06	2.66E+04	1.15E+04	1.15E+04
380	−5.38E+06	−5.38E+06	−6.13E+06	2.66E+04	1.27E+04	1.27E+04
385	−5.94E+06	−5.94E+06	−6.74E+06	2.66E+04	1.40E+04	1.40E+04
390	−6.54E+06	−6.54E+06	−7.40E+06	2.66E+04	1.53E+04	1.53E+04
395	−7.18E+06	−7.18E+06	−8.10E+06	2.66E+04	1.68E+04	1.68E+04
400	−7.88E+06	−7.88E+06	−8.86E+06	2.66E+04	1.84E+04	1.84E+04

**Table 10:** Variation in all normal and shear strain components with changes of thermal loading

T	$\varepsilon_{x}$	$\varepsilon_{y}$	$\varepsilon_{z}$	$\gamma_{xy}$	$\gamma_{xz}$	$\gamma_{yz}$
350	−5.6578E-07	−5.6578E-07	−6.68956E-06	1.13156E-06	2.83409E-07	2.83409E-07
355	−5.66226E-07	−5.6623E-07	−7.49836E-06	1.13245E-06	3.20347E-07	3.20347E-07
360	−5.67023E-07	−5.6702E-07	−8.32205E-06	1.13405E-06	3.5976E-07	3.5976E-07
365	−5.68091E-07	−5.6809E-07	−9.1611E-06	1.13618E-06	4.01898E-07	4.01898E-07
370	−5.69375E-07	−5.6938E-07	−1.0016E-05	1.13875E-06	4.47045E-07	4.47045E-07
375	−5.70833E-07	−5.7083E-07	−1.08871E-05	1.14167E-06	4.95531E-07	4.95531E-07
380	−5.72434E-07	−5.7243E-07	−1.1775E-05	1.14487E-06	5.47736E-07	5.47736E-07
385	−5.74153E-07	−5.7415E-07	−1.26801E-05	1.14831E-06	6.04102E-07	6.04102E-07
390	−5.7597E-07	−5.7597E-07	−1.3603E-05	1.15194E-06	6.65147E-07	6.65147E-07
395	−5.77868E-07	−5.7787E-07	−1.4544E-05	1.15574E-06	7.31477E-07	7.31477E-07
400	−5.79834E-07	−5.7983E-07	−1.55038E-05	1.15967E-06	8.03813E-07	8.03813E-07

Table 11 lists variation in various components of electric displacement and magnetic induction with changes in temperature rising. An enhancement in absolute value of all components of electric displacement and magnetic induction is observed with an increase in thermal loading.

**Table 11:** Variation in electric displacement and magnetic induction components with changes of thermal loading

T	$D_{x}$	$D_{y}$	$D_{z}$	$B_{x}$	$B_{y}$	$B_{z}$
350	1.64E-06	1.64E-06	−8.55E-05	7.79E-05	7.79E-05	−3.64E-03
355	1.86E-06	1.86E-06	−9.61E-05	8.81E-05	8.81E-05	−4.04E-03
360	2.09E-06	2.09E-06	−1.07E-04	9.89E-05	9.89E-05	−4.45E-03
365	2.33E-06	2.33E-06	−1.18E-04	1.11E-04	1.11E-04	−4.86E-03
370	2.59E-06	2.59E-06	−1.29E-04	1.23E-04	1.23E-04	−5.29E-03
375	2.87E-06	2.87E-06	−1.41E-04	1.36E-04	1.36E-04	−5.72E-03
380	3.18E-06	3.18E-06	−1.52E-04	1.51E-04	1.51E-04	−6.16E-03
385	3.50E-06	3.50E-06	−1.64E-04	1.66E-04	1.66E-04	−6.61E-03
390	3.86E-06	3.86E-06	−1.76E-04	1.83E-04	1.83E-04	−7.07E-03
395	4.24E-06	4.24E-06	−1.89E-04	2.01E-04	2.01E-04	−7.53E-03
400	4.66E-06	4.66E-06	−2.01E-04	2.21E-04	2.21E-04	−8.01E-03

## 5 Conclusions

Higher order electromagnetoelastic analysis of a shear deformable sandwich composite plate was presented in this paper based on principle of virtual work, Maxwell's equations and a thickness-stretchable model. The constitutive relations were extended in the Cartesian coordinate system for elastic core and piezoelectric/piezomagnetic face-sheets with accounting thermal strain and electromagnetic fields. The sandwich plate was composed of a graphene origami reinforced copper matrix core integrated with piezoelectric/piezomagnetic face-sheets subjected to thermoelectromagnetic loading. The kinematic relations were extended with assumption of bending, shear and stretching deformations. Effective modulus of elasticity, Poisson's ratio and thermal expansion coefficient were estimated using some modification coefficients in terms of folding degree and volume fraction of graphene origami and temperature. The parametric study is presented using the analytical solution to investigate impact of multi-field loading on the bending, shear and stretching deflections, maximum electric and magnetic potentials. The main conclusions of this paper are classified as follows:

An enhancement in $\bar{w_{b}}$ , $\bar{w_{s}}$ and stretching $\bar{\chi }$ is observed with an increase in $\Psi_{0}$ and $\Phi_{0}$.

The results show an increase in both maximum electric and magnetic potentials with an increase in $\Psi_{0}$ and $\Phi_{0}$.

Comparison between shear and stretching deflection rather than bending deflection indicates that this ratio becomes very important and comparable for thicker plates.

A decrease in both bending and shear deflections is observed with an increase in volume fraction of graphene origami. Furthermore, an increase in folding degree $H_{Gr}$ leads to an increase in absolute value of bending and shear deflections. One can conclude that an increase in folding degree leads to a decrease in structural stiffness of reinforced sandwich plate.

Investigating effect of folding and content of graphene yields a decrease in maximum electric and magnetic potentials with an increase in folding degree $H_{Gr}$. Furthermore, an increase in volume fractions $V_{Gr}$ leads to a decrease in ψ and φ.

## Data availability

No data was used for the research described in the article.

## CRediT authorship contribution statement

**Thaier J. Ntayeesh:** Writing – original draft, Software, Funding acquisition, Conceptualization. **Mohammad Arefi:** Writing – original draft, Visualization, Validation, Project administration, Methodology, Formal analysis, Data curation, Conceptualization.

## Declaration of competing interest

The authors declare that they have no known competing financial interests or personal relationships that could have appeared to influence the work reported in this paper.

## Appendix A

$$\left\{J_{1},J_{2},J_{3}\right\}=\int_{-\frac{h}{2}-h_{p}}^{-\frac{h}{2}}C_{11}^{P}\left\{1,z,f\left(z\right)\right\}dz+\int_{-\frac{h}{2}}^{\frac{h}{2}}\frac{E\left(1-\vartheta \right)}{\left(1-2\vartheta \right)\left(1+\vartheta \right)}\left\{1,z,f\left(z\right)\right\}dz+\int_{\frac{h}{2}}^{\frac{h}{2}+h_{p}}C_{11}^{P}\left\{1,z,f\left(z\right)\right\}dz\text{,}$$

$$\left\{J_{4},J_{5},J_{6}\right\}=\int_{-\frac{h}{2}-h_{p}}^{-\frac{h}{2}}C_{12}^{P}\left\{1,z,f\left(z\right)\right\}dz+\int_{-\frac{h}{2}}^{\frac{h}{2}}\frac{E\vartheta }{\left(1-2\vartheta \right)\left(1+\vartheta \right)}\left\{1,z,f\left(z\right)\right\}dz+\int_{\frac{h}{2}}^{\frac{h}{2}+h_{p}}C_{12}^{P}\left\{1,z,f\left(z\right)\right\}dz$$

$$\left\{J_{7}\right\}=\int_{-\frac{h}{2}-h_{p}}^{-\frac{h}{2}}C_{13}^{P}g^{\prime }\left(z\right)dz+\int_{-\frac{h}{2}}^{\frac{h}{2}}\frac{E\vartheta g^{\prime }\left(z\right)}{\left(1-2\vartheta \right)\left(1+\vartheta \right)}dz+\int_{\frac{h}{2}}^{\frac{h}{2}+h_{p}}C_{13}^{P}g^{\prime }\left(z\right)dz$$

$$\left\{R_{1},R_{2}\right\}=\int_{-\frac{h}{2}-h_{p}}^{-\frac{h}{2}}\frac{\pi }{h}\left\{e_{31},q_{31}\right\}\sin\frac{\pi z}{h}dz+\int_{\frac{h}{2}}^{\frac{h}{2}+h_{p}}\frac{\pi }{h}\left\{e_{31},q_{31}\right\}\sin\frac{\pi z}{h}dz\text{,}$$

$$\left\{N_{xx}^{\Psi_{0}},N_{xx}^{\Phi_{0}}\right\}=\int_{-\frac{h}{2}-h_{p}}^{-\frac{h}{2}}\frac{2}{h}\left\{e_{31}\Psi_{0},q_{31}\Phi_{0}\right\}\sin\frac{\pi z}{h}dz+\int_{\frac{h}{2}}^{\frac{h}{2}+h_{p}}\frac{2}{h}\left\{e_{31}\Psi_{0},q_{31}\Phi_{0}\right\}\sin\frac{\pi z}{h}dz\text{,}$$

$$\left\{J_{1}^{T},J_{2}^{T},J_{3}^{T},J_{4}^{T}\right\}=\int_{-\frac{h}{2}-h_{p}}^{-\frac{h}{2}}\left(C_{13}^{P}+C_{12}^{P}+C_{11}^{P}\right)\alpha \left\{1,z,f\left(z\right),\frac{dg\left(z\right)}{dz}\right\}dz+\int_{-\frac{h}{2}}^{\frac{h}{2}}\frac{E\alpha }{\left(1-2\vartheta \right)}\alpha \left\{1,z,f\left(z\right),\frac{dg\left(z\right)}{dz}\right\}dz+\int_{\frac{h}{2}}^{\frac{h}{2}+h_{p}}\left(C_{13}^{P}+C_{12}^{P}+C_{11}^{P}\right)\alpha \left\{1,z,f\left(z\right),\frac{dg\left(z\right)}{dz}\right\}dz$$

$$\left\{R_{7},R_{8}\right\}=\int_{-\frac{h}{2}-h_{p}}^{-\frac{h}{2}}\frac{\pi }{h}\left\{e_{32},q_{32}\right\}\sin\frac{\pi z}{h}dz+\int_{\frac{h}{2}}^{\frac{h}{2}+h_{p}}\frac{\pi }{h}\left\{e_{32},q_{32}\right\}\sin\frac{\pi z}{h}dz\text{,}$$

$$\left\{N_{yy}^{\Psi_{0}},N_{yy}^{\Phi_{0}}\right\}=\int_{-\frac{h}{2}-h_{p}}^{-\frac{h}{2}}\frac{2}{h}\left\{e_{32}\Psi_{0},q_{32}\Phi_{0}\right\}\sin\frac{\pi z}{h}dz+\int_{\frac{h}{2}}^{\frac{h}{2}+h_{p}}\frac{2}{h}\left\{e_{32}\Psi_{0},q_{32}\Phi_{0}\right\}\sin\frac{\pi z}{h}dz\text{,}$$

$$\left\{R_{9},R_{10}\right\}=\int_{-\frac{h}{2}-h_{p}}^{-\frac{h}{2}}\frac{\pi }{h}z\left\{e_{32},q_{32}\right\}\sin\frac{\pi z}{h}dz+\int_{\frac{h}{2}}^{\frac{h}{2}+h_{p}}\frac{\pi }{h}z\left\{e_{32},q_{32}\right\}\sin\frac{\pi z}{h}dz\text{,}$$

$$\left\{M_{yy}^{\Psi_{0}},M_{yy}^{\Phi_{0}}\right\}=\int_{-\frac{h}{2}-h_{p}}^{-\frac{h}{2}}\frac{2}{h}z\left\{e_{32}\Psi_{0},q_{32}\Phi_{0}\right\}\sin\frac{\pi z}{h}dz+\int_{\frac{h}{2}}^{\frac{h}{2}+h_{p}}\frac{2}{h}z\left\{e_{32}\Psi_{0},q_{32}\Phi_{0}\right\}\sin\frac{\pi z}{h}dz\text{,}$$

$$\left\{J_{8},J_{9},J_{10}\right\}=\int_{-\frac{h}{2}-h_{p}}^{-\frac{h}{2}}C_{11}^{P}z\left\{1,z,f\left(z\right)\right\}dz+\int_{-\frac{h}{2}}^{\frac{h}{2}}\frac{E\left(1-\vartheta \right)}{\left(1-2\vartheta \right)\left(1+\vartheta \right)}z\left\{1,z,f\left(z\right)\right\}dz+\int_{\frac{h}{2}}^{\frac{h}{2}+h_{p}}C_{11}^{P}z\left\{1,z,f\left(z\right)\right\}dz\text{,}$$

$$\left\{J_{11},J_{12},J_{13}\right\}=\int_{-\frac{h}{2}-h_{p}}^{-\frac{h}{2}}C_{12}^{P}z\left\{1,z,f\left(z\right)\right\}dz+\int_{-\frac{h}{2}}^{\frac{h}{2}}\frac{E\vartheta }{\left(1-2\vartheta \right)\left(1+\vartheta \right)}z\left\{1,z,f\left(z\right)\right\}dz+\int_{\frac{h}{2}}^{\frac{h}{2}+h_{p}}C_{12}^{P}z\left\{1,z,f\left(z\right)\right\}dz\text{,}$$

$$\left\{J_{14}\right\}=\int_{-\frac{h}{2}-h_{p}}^{-\frac{h}{2}}C_{13}^{P}zg^{\prime }\left(z\right)dz+\int_{-\frac{h}{2}}^{\frac{h}{2}}\frac{E\vartheta g^{\prime }\left(z\right)}{\left(1-2\vartheta \right)\left(1+\vartheta \right)}zdz+\int_{\frac{h}{2}}^{\frac{h}{2}+h_{p}}C_{13}^{P}zg^{\prime }\left(z\right)dz$$

$$\left\{R_{3},R_{4}\right\}=\int_{-\frac{h}{2}-h_{p}}^{-\frac{h}{2}}\frac{\pi }{h}z\left\{e_{31},q_{31}\right\}\sin\frac{\pi z}{h}dz+\int_{\frac{h}{2}}^{\frac{h}{2}+h_{p}}\frac{\pi }{h}z\left\{e_{31},q_{31}\right\}\sin\frac{\pi z}{h}dz\text{,}$$

$$\left\{M_{xx}^{\Psi_{0}},M_{xx}^{\Phi_{0}}\right\}=\int_{-\frac{h}{2}-h_{p}}^{-\frac{h}{2}}\frac{2}{h}z\left\{e_{31}\Psi_{0},q_{31}\Phi_{0}\right\}\sin\frac{\pi z}{h}dz+\int_{\frac{h}{2}}^{\frac{h}{2}+h_{p}}\frac{2}{h}z\left\{e_{31}\Psi_{0},q_{31}\Phi_{0}\right\}\sin\frac{\pi z}{h}dz\text{,}$$

$$\left\{R_{11},R_{12}\right\}=\int_{-\frac{h}{2}-h_{p}}^{-\frac{h}{2}}\frac{\pi }{h}f\left(zZ\right)\left\{e_{32},q_{32}\right\}\sin\frac{\pi z}{h}dz+\int_{\frac{h}{2}}^{\frac{h}{2}+h_{p}}\frac{\pi }{h}f\left(z\right)\left\{e_{32},q_{32}\right\}\sin\frac{\pi z}{h}dz\text{,}$$

$$\left\{S_{yy}^{\Psi_{0}},S_{yy}^{\Phi_{0}}\right\}=\int_{-\frac{h}{2}-h_{p}}^{-\frac{h}{2}}\frac{2}{h}f\left(z\right)\left\{e_{32}\Psi_{0},q_{32}\Phi_{0}\right\}\sin\frac{\pi z}{h}dz+\int_{\frac{h}{2}}^{\frac{h}{2}+h_{p}}\frac{2}{h}f\left(z\right)\left\{e_{32}\Psi_{0},q_{32}\Phi_{0}\right\}\sin\frac{\pi z}{h}\text{dz,}$$

$$\left\{J_{15},J_{16},J_{17}\right\}=\int_{-\frac{h}{2}-h_{p}}^{-\frac{h}{2}}C_{11}^{P}f\left(z\right)\left\{1,z,f\left(z\right)\right\}dz+\int_{-\frac{h}{2}}^{\frac{h}{2}}\frac{E\left(1-\vartheta \right)}{\left(1-2\vartheta \right)\left(1+\vartheta \right)}f\left(z\right)\left\{1,z,f\left(z\right)\right\}dz+\int_{\frac{h}{2}}^{\frac{h}{2}+h_{p}}C_{11}^{P}f\left(z\right)\left\{1,z,f\left(z\right)\right\}dz\text{,}$$

$$\left\{J_{18},J_{19},J_{20}\right\}=\int_{-\frac{h}{2}-h_{p}}^{-\frac{h}{2}}C_{12}^{P}f\left(z\right)\left\{1,z,f\left(z\right)\right\}dz+\int_{-\frac{h}{2}}^{\frac{h}{2}}\frac{E\vartheta }{\left(1-2\vartheta \right)\left(1+\vartheta \right)}f\left(z\right)\left\{1,z,f\left(z\right)\right\}dz+\int_{\frac{h}{2}}^{\frac{h}{2}+h_{p}}C_{12}^{P}f\left(z\right)\left\{1,z,f\left(z\right)\right\}dz\text{,}$$

$$\left\{J_{21}\right\}=\int_{-\frac{h}{2}-h_{p}}^{-\frac{h}{2}}C_{13}^{P}f\left(z\right)g^{\prime }\left(z\right)dz+\int_{-\frac{h}{2}}^{\frac{h}{2}}\frac{E\vartheta g^{\prime }\left(z\right)}{\left(1-2\vartheta \right)\left(1+\vartheta \right)}f\left(z\right)dz+\int_{\frac{h}{2}}^{\frac{h}{2}+h_{p}}C_{13}^{P}f\left(z\right)g^{\prime }\left(z\right)dz$$

$$\left\{R_{5},R_{6}\right\}=\int_{-\frac{h}{2}-h_{p}}^{-\frac{h}{2}}\frac{\pi }{h}f\left(z\right)\left\{e_{31},q_{31}\right\}\sin\frac{\pi z}{h}dz+\int_{\frac{h}{2}}^{\frac{h}{2}+h_{p}}\frac{\pi }{h}f\left(z\right)\left\{e_{31},q_{31}\right\}\sin\frac{\pi z}{h}dz\text{,}$$

$$\left\{S_{xx}^{\Psi_{0}},S_{xx}^{\Phi_{0}}\right\}=\int_{-\frac{h}{2}-h_{p}}^{-\frac{h}{2}}\frac{2}{h}f\left(z\right)\left\{e_{31}\Psi_{0},q_{31}\Phi_{0}\right\}\sin\frac{\pi z}{h}dz+\int_{\frac{h}{2}}^{\frac{h}{2}+h_{p}}\frac{2}{h}f\left(z\right)\left\{e_{31}\Psi_{0},q_{31}\Phi_{0}\right\}\sin\frac{\pi z}{h}dz\text{,}$$

$$\left\{J_{22},J_{23},J_{24}\right\}=\int_{-\frac{h}{2}-h_{p}}^{-\frac{h}{2}}C_{66}^{P}\left\{1,z,f\left(z\right)\right\}dz+\int_{-\frac{h}{2}}^{\frac{h}{2}}\frac{E}{\left(1-2\vartheta \right)\left(1+\vartheta \right)}\frac{1-2\vartheta }{2}\left\{1,z,f\left(z\right)\right\}dz+\int_{\frac{h}{2}}^{\frac{h}{2}+h_{p}}C_{66}^{P}\left\{1,z,f\left(z\right)\right\}dz\text{,}$$

$$\left\{J_{25},J_{26},J_{27}\right\}=\int_{-\frac{h}{2}-h_{p}}^{-\frac{h}{2}}C_{66}^{P}z\left\{1,z,f\left(z\right)\right\}dz+\int_{-\frac{h}{2}}^{\frac{h}{2}}\frac{E}{\left(1-2\vartheta \right)\left(1+\vartheta \right)}\frac{1-2\vartheta }{2}z\left\{1,z,f\left(z\right)\right\}dz+\int_{\frac{h}{2}}^{\frac{h}{2}+h_{p}}C_{66}^{P}z\left\{1,z,f\left(z\right)\right\}dz$$

$$\left\{J_{28},J_{29},J_{30}\right\}=\int_{-\frac{h}{2}-h_{p}}^{-\frac{h}{2}}C_{66}^{P}f\left(z\right)\left\{1,z,f\left(z\right)\right\}dz+\int_{-\frac{h}{2}}^{\frac{h}{2}}\frac{E}{\left(1-2\vartheta \right)\left(1+\vartheta \right)}\frac{1-2\vartheta }{2}f\left(z\right)\left\{1,z,f\left(z\right)\right\}dz+\int_{\frac{h}{2}}^{\frac{h}{2}+h_{p}}C_{66}^{P}f\left(z\right)\left\{1,z,f\left(z\right)\right\}dz\text{,}$$

$$\left\{R_{13},R_{14}\right\}=\int_{-\frac{h}{2}-h_{p}}^{-\frac{h}{2}}\frac{\pi }{h}g^{\prime }\left(z\right)\left\{e_{33},q_{33}\right\}\sin\frac{\pi z}{h}dz+\int_{\frac{h}{2}}^{\frac{h}{2}+h_{p}}\frac{\pi }{h}g^{\prime }\left(z\right)\left\{e_{33},q_{33}\right\}\sin\frac{\pi z}{h}dz\text{,}$$

$$\left\{N_{zz}^{\Psi_{0}},N_{zz}^{\Phi_{0}}\right\}=\int_{-\frac{h}{2}-h_{p}}^{-\frac{h}{2}}\frac{2}{h}g^{\prime }\left(z\right)\left\{e_{33}\Psi_{0},q_{33}\Phi_{0}\right\}\sin\frac{\pi z}{h}dz+\int_{\frac{h}{2}}^{\frac{h}{2}+h_{p}}\frac{2}{h}g^{\prime }\left(z\right)\left\{e_{33}\Psi_{0},q_{33}\Phi_{0}\right\}\sin\frac{\pi z}{h}dz\text{,}$$

$$\left\{J_{31},J_{32},J_{33}\right\}=\int_{-\frac{h}{2}-h_{p}}^{-\frac{h}{2}}C_{13}^{P}g^{\prime }\left(z\right)\left\{1,z,f\left(z\right)\right\}dz+\int_{-\frac{h}{2}}^{\frac{h}{2}}\frac{E\vartheta }{\left(1-2\vartheta \right)\left(1+\vartheta \right)}g^{\prime }\left(z\right)\left\{1,z,f\left(z\right)\right\}dz+\int_{\frac{h}{2}}^{\frac{h}{2}+h_{p}}C_{13}^{P}g^{\prime }\left(z\right)\left\{1,z,f\left(z\right)\right\}dz\text{,}$$

$$\left\{J_{34},J_{35},J_{36}\right\}=\int_{-\frac{h}{2}-h_{p}}^{-\frac{h}{2}}C_{23}^{P}g^{\prime }\left(z\right)\left\{1,z,f\left(z\right)\right\}dz+\int_{-\frac{h}{2}}^{\frac{h}{2}}\frac{E\vartheta }{\left(1-2\vartheta \right)\left(1+\vartheta \right)}g^{\prime }\left(z\right)\left\{1,z,f\left(z\right)\right\}dz+\int_{\frac{h}{2}}^{\frac{h}{2}+h_{p}}C_{23}^{P}g^{\prime }\left(z\right)\left\{1,z,f\left(z\right)\right\}dz\text{,}$$

$$\left\{J_{37}\right\}=\int_{-\frac{h}{2}-h_{p}}^{-\frac{h}{2}}C_{33}^{P}g^{\prime }\left(z\right)g^{\prime }\left(z\right)dz+\int_{-\frac{h}{2}}^{\frac{h}{2}}\frac{E\left(1-\vartheta \right)g^{\prime }\left(z\right)}{\left(1-2\vartheta \right)\left(1+\vartheta \right)}g^{\prime }\left(z\right)dz+\int_{\frac{h}{2}}^{\frac{h}{2}+h_{p}}C_{33}^{P}g^{\prime }\left(z\right)g^{\prime }\left(z\right)dz$$

$$\left\{J_{38}\right\}=\int_{-\frac{h}{2}-h_{p}}^{-\frac{h}{2}}C_{55}^{P}g^{2}\left(z\right)dz+\int_{-\frac{h}{2}}^{\frac{h}{2}}\frac{E}{\left(1-2\vartheta \right)\left(1+\vartheta \right)}\frac{1-2\vartheta }{2}g^{2}\left(z\right)\text{dz}+\int_{\frac{h}{2}}^{\frac{h}{2}+h_{p}}C_{55}^{P}g^{2}\left(z\right)dz$$

$$\left\{J_{39}\right\}=\int_{-\frac{h}{2}-h_{p}}^{-\frac{h}{2}}C_{44}^{P}g^{2}\left(z\right)dz+\int_{-\frac{h}{2}}^{\frac{h}{2}}\frac{E}{\left(1-2\vartheta \right)\left(1+\vartheta \right)}\frac{1-2\vartheta }{2}g^{2}\left(z\right)dz+\int_{\frac{h}{2}}^{\frac{h}{2}+h_{p}}C_{44}^{P}g^{2}\left(z\right)dz$$

$$\left\{R_{15},R_{16},R_{17},R_{18}\right\}=\int_{-\frac{h}{2}-h_{p}}^{-\frac{h}{2}}g^{2}\left(z\right)\left\{e_{15},q_{15},e_{24},q_{24}\right\}\cos\frac{\pi z}{h}dz+\int_{\frac{h}{2}}^{\frac{h}{2}+h_{p}}g^{2}\left(z\right)\left\{e_{15},q_{15},e_{24},q_{24}\right\}\cos\frac{\pi z}{h}dz\text{,}$$

$$\left\{J_{5}^{T}\right\}=\int_{-\frac{h}{2}-h_{p}}^{-\frac{h}{2}}\frac{\pi }{h}\sin\frac{\pi z}{h}\alpha_{eff}\left(e_{31}+e_{32}+e_{33}\right)dz+\int_{\frac{h}{2}}^{\frac{h}{2}+h_{p}}\frac{\pi }{h}\sin\frac{\pi z}{h}\alpha_{eff}\left(e_{31}+e_{32}+e_{33}\right)dz$$

$$\left\{R_{32},R_{33}\right\}=\int_{-\frac{h}{2}-h_{p}}^{-\frac{h}{2}}{\left(\frac{\pi }{h}\sin\frac{\pi z}{h}\right)}^{2}\left\{\eta_{33},d_{33}\right\}dz+\int_{\frac{h}{2}}^{\frac{h}{2}+h_{p}}{\left(\frac{\pi }{h}\sin\frac{\pi z}{h}\right)}^{2}\left\{\eta_{33},d_{33}\right\}dz$$

$$\left\{D_{z}^{\Psi_{0}},D_{z}^{\Phi_{0}}\right\}=\int_{-\frac{h}{2}-h_{p}}^{-\frac{h}{2}}\frac{\pi }{h}\frac{2}{h}\sin\frac{\pi z}{h}\left\{\eta_{33}\Psi_{0},d_{33}\Phi_{0}\right\}dz+\int_{\frac{h}{2}}^{\frac{h}{2}+h_{p}}\frac{\pi }{h}\frac{2}{h}\sin\frac{\pi z}{h}\left\{\eta_{33}\Psi_{0},d_{33}\Phi_{0}\right\}dz$$

$$\left\{R_{19},R_{20}\right\}=\int_{-\frac{h}{2}-h_{p}}^{-\frac{h}{2}}g\left(z\right)\cos\frac{\pi z}{h}\left\{e_{15},e_{24}\right\}dz+\int_{\frac{h}{2}}^{\frac{h}{2}+h_{p}}g\left(z\right)\cos\frac{\pi z}{h}\left\{e_{15},e_{24}\right\}dz$$

$$\left\{R_{21},R_{22},R_{23},R_{24}\right\}=\int_{-\frac{h}{2}-h_{p}}^{-\frac{h}{2}}\;\cos^{2}\frac{\pi z}{h}\left\{\eta_{11},d_{11},\eta_{22},d_{22}\right\}dz+\int_{\frac{h}{2}}^{\frac{h}{2}+h_{p}}\;\cos^{2}\frac{\pi z}{h}\left\{\eta_{11},d_{11},\eta_{22},d_{22}\right\}dz\text{,}$$

$$\left\{R_{25},R_{26},R_{27}\right\}=\int_{-\frac{h}{2}-h_{p}}^{-\frac{h}{2}}\frac{\pi }{h}\sin\frac{\pi z}{h}e_{31}\left\{1,z,f\left(z\right)\right\}dz+\int_{\frac{h}{2}}^{\frac{h}{2}+h_{p}}\frac{\pi }{h}\sin\frac{\pi z}{h}e_{31}\left\{1,z,f\left(z\right)\right\}dz$$

$$\left\{R_{28},R_{29},R_{30}\right\}=\int_{-\frac{h}{2}-h_{p}}^{-\frac{h}{2}}\frac{\pi }{h}\sin\frac{\pi z}{h}e_{32}\left\{1,z,f\left(z\right)\right\}dz+\int_{\frac{h}{2}}^{\frac{h}{2}+h_{p}}\frac{\pi }{h}\sin\frac{\pi z}{h}e_{32}\left\{1,z,f\left(z\right)\right\}dz$$

$$\left\{R_{31}\right\}=\int_{-\frac{h}{2}-h_{p}}^{-\frac{h}{2}}\frac{\pi }{h}\sin\frac{\pi z}{h}e_{33}\frac{dg\left(z\right)}{dz}dz+\int_{\frac{h}{2}}^{\frac{h}{2}+h_{p}}\frac{\pi }{h}\sin\frac{\pi z}{h}e_{33}\frac{dg\left(z\right)}{dz}dz$$

$$\left\{J_{5}^{T}\right\}=\int_{-\frac{h}{2}-h_{p}}^{-\frac{h}{2}}\frac{\pi }{h}\sin\frac{\pi z}{h}\alpha_{eff}\left(q_{31}+q_{32}+q_{33}\right)dz+\int_{\frac{h}{2}}^{\frac{h}{2}+h_{p}}\frac{\pi }{h}\sin\frac{\pi z}{h}\alpha_{eff}\left(q_{31}+q_{32}+q_{33}\right)dz$$

$$\left\{R_{47},R_{48}\right\}=\int_{-\frac{h}{2}-h_{p}}^{-\frac{h}{2}}{\left(\frac{\pi }{h}\sin\frac{\pi z}{h}\right)}^{2}\left\{d_{33},\mu_{33}\right\}dz+\int_{\frac{h}{2}}^{\frac{h}{2}+h_{p}}{\left(\frac{\pi }{h}\sin\frac{\pi z}{h}\right)}^{2}\left\{d_{33},\mu_{33}\right\}dz$$

$$\left\{B_{z}^{\Psi_{0}},B_{z}^{\Phi_{0}}\right\}=\int_{-\frac{h}{2}-h_{p}}^{-\frac{h}{2}}\frac{\pi }{h}\frac{2}{h}\sin\frac{\pi z}{h}\left\{d_{33}\Psi_{0},\mu_{33}\Phi_{0}\right\}dz+\int_{\frac{h}{2}}^{\frac{h}{2}+h_{p}}\frac{\pi }{h}\frac{2}{h}\sin\frac{\pi z}{h}\left\{d_{33}\Psi_{0},{d\mu }_{33}\Phi_{0}\right\}dz$$

$$\left\{R_{34},R_{35}\right\}=\int_{-\frac{h}{2}-h_{p}}^{-\frac{h}{2}}g\left(z\right)\cos\frac{\pi z}{h}\left\{q_{15},q_{24}\right\}dz+\int_{\frac{h}{2}}^{\frac{h}{2}+h_{p}}g\left(z\right)\cos\frac{\pi z}{h}\left\{q_{15},q_{24}\right\}dz$$

$$\left\{R_{36},R_{37},R_{38},R_{39}\right\}=\int_{-\frac{h}{2}-h_{p}}^{-\frac{h}{2}}\;\cos^{2}\frac{\pi z}{h}\left\{d_{11},\mu_{11},d_{22},\mu_{22}\right\}dz+\int_{\frac{h}{2}}^{\frac{h}{2}+h_{p}}\;\cos^{2}\frac{\pi z}{h}\left\{d_{11},\mu_{11},d_{22},\mu_{22}\right\}dz\text{,}$$

$$\left\{R_{40},R_{41},R_{42}\right\}=\int_{-\frac{h}{2}-h_{p}}^{-\frac{h}{2}}\frac{\pi }{h}\sin\frac{\pi z}{h}q_{31}\left\{1,z,f\left(z\right)\right\}dz+\int_{\frac{h}{2}}^{\frac{h}{2}+h_{p}}\frac{\pi }{h}\sin\frac{\pi z}{h}q_{31}\left\{1,z,f\left(z\right)\right\}dz$$

$$\left\{R_{43},R_{44},R_{45}\right\}=\int_{-\frac{h}{2}-h_{p}}^{-\frac{h}{2}}\frac{\pi }{h}\sin\frac{\pi z}{h}q_{32}\left\{1,z,f\left(z\right)\right\}dz+\int_{\frac{h}{2}}^{\frac{h}{2}+h_{p}}\frac{\pi }{h}\sin\frac{\pi z}{h}q_{32}\left\{1,z,f\left(z\right)\right\}dz\text{,}$$

$$\left\{R_{46}\right\}=\int_{-\frac{h}{2}-h_{p}}^{-\frac{h}{2}}\frac{\pi }{h}\sin\frac{\pi z}{h}q_{33}\frac{dg\left(z\right)}{dz}dz+\int_{\frac{h}{2}}^{\frac{h}{2}+h_{p}}\frac{\pi }{h}\sin\frac{\pi z}{h}q_{33}\frac{dg\left(z\right)}{dz}dz$$
